# Rethinking Giftedness in the Age of Generative AI: A Relational Epistemic Agency Model for Advanced Learners

**DOI:** 10.3390/jintelligence14100247

**Published:** 2026-10-07

**Authors:** Çiğdem Nilüfer Umar

**Affiliations:** Department of Special Education, Faculty of Education, Çanakkale Onsekiz Mart University, 17100 Çanakkale, Turkey; umar@comu.edu.tr

**Keywords:** advanced learners, giftedness, generative artificial intelligence, epistemic agency, cognitive offloading, intellectual ownership, transfer, assessment validity

## Abstract

Generative artificial intelligence can enhance the quality and visibility of advanced learners’ work while making it harder to determine what the resulting performance reveals about their independent competence. This theoretical paper develops the Relational Epistemic Agency Model for Advanced Learners (REAM-AL) through a structured database search and an integrative synthesis of scholarship on talent development, extended and distributed cognition, cognitive offloading, metacognition, epistemic agency, intellectual ownership, transfer, and assessment validity. The model organizes five focal constructs across four analytical layers: developmental epistemic readiness, cognitive task distribution, epistemic decision distribution, intellectual ownership, and the relationship among supported performance, independent competence, and transfer. Their configuration may produce relational cognitive extension, in which AI broadens thinking while epistemic authority remains with the learner, or developmental substitution, in which the system performs operations the learner is expected to develop. REAM-AL treats these as interaction patterns rather than fixed student categories. It offers a framework for task design and assessment that distinguishes joint human–AI performance from independently sustained and transferable competence. The proposed constructs and relationships remain theoretical and require direct testing with advanced children and adolescents across varied tasks, developmental periods, and AI conditions.

## 1. Introduction

Generative AI can make an advanced learner’s capabilities more visible, but it can also blur the extent to which the resulting performance belongs to the student. With AI support, a student may solve a complex problem, develop a seemingly original product, or produce a text well beyond what would normally be expected at that age. Yet the quality of the final product does not, in itself, reveal who framed the problem, selected the evidence, or constructed the reasoning. The central issue, then, is not simply whether students perform better with technology. What matters is how that performance was produced and what, if anything, it allows us to infer about their development.

Developmental theories of giftedness help explain why this distinction matters. From this perspective, giftedness cannot be reduced to a high intelligence score obtained at a particular point in time or to the production of an exceptional outcome. Rather, it is understood as a dynamic process that unfolds through the continuing interaction between the individual and the environment (Dai, 2017; Subotnik et al., 2011). The transformation of initial cognitive potential into domain-specific competence and creative productivity depends on the combined influence of systematic instruction, domain knowledge and skills, motivation, psychosocial characteristics, and access to high-quality developmental opportunities (Dai, 2017; Subotnik et al., 2011). When this developmental view is applied to generative AI, a crucial distinction emerges: Does technology support students in making more effective use of capabilities they already possess, or does it perform on their behalf a competence they have yet to develop? This distinction is essential both for tracking the development of advanced learners and for making valid judgments about the performance they demonstrate.

For gifted students and other advanced learners, generative AI opens several plausible educational possibilities. It may support personalization, differentiation, independent inquiry, writing, and mentoring, although the direct evidence remains limited and is discussed in Section 2.1 (Almumen & Jouhar, 2025; Guilbault et al., 2025). These early findings do not yet establish that comparable benefits extend to advanced children and adolescents or persist once AI support is no longer available.

Evidence in this area remains preliminary. Alsamani and Alsamiri’s (2026) review of 26 studies found marked variation in research methods, participant ages, educational levels, AI systems, and outcomes. Much of the work was small in scale, exploratory, or limited to short interventions. We now know more about how generative AI might be used for personalization, writing, creativity, question generation, and mentoring than we do about what learners can sustain when AI no longer performs the target operation. Claims that these tools strengthen the independent talent development of advanced learners would therefore be premature.

For advanced learners, this gap in the evidence is more than a routine measurement problem. A student with strong domain knowledge, verbal fluency, and a capacity to grasp ideas quickly may select apparently relevant material from AI-generated responses and shape it into a sophisticated, coherent, and persuasive product. The quality of that product, however, does not necessarily mean that the student framed the problem, formulated the central claim, or developed the underlying line of reasoning. Strong expressive and organizational skills may make the final product appear to be an independent accomplishment even when AI has carried out much of the thinking at the heart of the task. Judging advanced competence solely from product quality may therefore conceal a possible gap between what students can produce with AI and what they are actually developing.

Evidence from general university samples also indicates that improvements in AI-supported products do not necessarily coincide with equivalent gains in knowledge, retention, or transfer (Barcaui, 2025; Fan et al., 2025). Although these findings cannot be generalized directly to advanced children, they establish the need to measure joint performance and learning outcomes separately.

Creativity raises a parallel, though slightly different, problem. Generative AI can place many ideas and possibilities before an advanced learner in a matter of seconds. In general adult samples, Lee and Chung (2024) found that ideas produced with ChatGPT received higher creativity ratings than ideas generated without technological support or through conventional web search. Meincke et al. (2025), reanalyzing Lee and Chung’s openly shared data, found less diversity across the full pool of ChatGPT-assisted ideas than across the comparison pools. An individual response may therefore look more creative even as the collective range of ideas narrows. For advanced learners, this means that creativity judgment cannot rest on the final product alone. The student’s role in choosing the problem, combining and transforming suggestions, and explaining why a particular idea was pursued also matters. Neither study examined advanced children or adolescents, and neither followed creative development over time; conclusions about enduring growth must remain tentative.

Cognitive offloading helps explain why a stronger product does not need to signal equivalent growth in the learner. External tools can lower the immediate demands of a task and free limited internal resources for other operations, whether carried out at the same time or later (Grinschgl & Neubauer, 2022; Risko & Gilbert, 2016). Offloading is not inherently a developmental loss. Generative AI does, however, differ from tools used mainly to store, retrieve, or remember information. It can participate directly in academic writing, information seeking, and problem solving. What is offloaded may consequently include not only memory and routine operations but also cognitive and metacognitive processes that the student is expected to acquire (Grinschgl & Neubauer, 2022; Yan et al., 2025). This remains a theoretical possibility, not an established developmental effect. It arises from reading the cognitive-offloading evidence alongside the newer work on generative AI.

The amount of work handed to AI is less informative than the nature and purpose of that work. A student may ask for a counterexample, invite criticism of a proposed solution, or request an alternative explanation. If those suggestions are questioned, filtered, and used to build more complex relationships, offloading can create space for further thought. A different developmental situation arises when AI repeatedly frames the problem, selects the evidence, constructs the justification, or draws the conclusion even though those processes are central to the learning goal. The product may look successful while the student’s independent competence changes very little. Here, that second pattern is termed developmental substitution, a concept derived from the intersection of the cognitive-offloading and performance–learning literature.

Students cannot be expected to locate the boundary between strategic support and developmental substitution accurately in every task. Metacognitive monitoring and control change considerably during childhood, and children do not always deploy them spontaneously or successfully. They may overestimate what they know, misread the difficulty of a task, or choose support that does not match their actual needs (Bayard et al., 2021; O’Leary & Sloutsky, 2017). Evidence from university students illustrates the underlying mechanism. Urban et al. (2025) found that ChatGPT support increased the amount of correct information incorporated into written work without significantly reducing the uptake of false information generated by the system. Xu et al. (2025) reported no significant between-group achievement difference following explicit metacognitive support, yet students improved in task strategy, self-evaluation, and self-regulated learning. Access, in other words, is only part of the educational arrangement; the interaction itself must be structured with the learner’s developmental profile and metacognitive needs in mind. Because both studies involved university students, they clarify a possible mechanism rather than provide direct evidence about advanced children and adolescents.

Gifted identification alone does not ensure that interaction with generative AI becomes higher-order inquiry. The limited target-population evidence suggests substantial variation in how gifted students question and use AI, reinforcing the need to distinguish advanced cognitive potential from epistemic readiness (Kang & Lee, 2023).

Metacognitive control sheds light on how students monitor and regulate their thinking. It says less about who retains authority over the knowledge-related decisions made during an AI-supported exchange. That question belongs to epistemic agency: the learner’s deliberate participation in building knowledge, active role in judging ideas and evidence, and responsibility for moving an inquiry forward (Damşa et al., 2010; González-Howard & McNeill, 2020; Scardamalia, 2002).

In human–AI work, visible activity can be misleading. A student may write many prompts, sustain a long exchange, and heavily edit the generated text without retaining authority over the inquiry. Prompting demonstrates some degree of tool competence; it does not show who decided how the problem should be framed, which evidence mattered, what method should be followed, or which conclusion deserved support. Epistemic agency is therefore located in the responsibility students take for those decisions: framing the problem, evaluating ideas and evidence, participating in methodological choices, and defending the conclusion they endorse.

A distinction must therefore be drawn between what AI does for the task and who holds epistemic authority over it. The system may retrieve information, generate examples and options, compare alternatives, or revise prose. These can be substantial contributions, but their size does not entitle the system to set the educational purpose of the task or carry responsibility for the resulting claims. Responsibility remains visible when the student can explain, justify, and defend the decisions through which the work was produced. Within REAM-AL, the distribution of epistemic decision-making captures how authority over problem framing, method selection, evidence evaluation, the central claim, and the final conclusion moves among the student, AI, and, where relevant, the teacher. This shifts attention from how often AI was used to the decision structure of the knowledge-building process.

How those decisions are distributed also bears directly on intellectual ownership and assessment validity. Intellectual ownership, as used here, is evident when students understand the product’s central ideas and reasoning, can separate their contribution from the system’s, and can defend the conclusions with appropriate evidence. It is not equivalent to legal authorship, a count of human-written words, or disclosure of AI use. Ebrahimzadeh et al. (2026) identify a threat to coauthorship integrity and assessment validity when students present AI-generated material they do not understand as their own, and they call for assessment designs that verify students’ epistemic ownership of their writing. The origin of the prose is only one part of that judgment. The student’s understanding, reasoning, and capacity to defend the work are equally important.

Knowing whether prose is likely to be human- or AI-generated tells us little, by itself, about the student’s understanding. Experienced assessors in Kofinas et al. (2025) could not reliably distinguish work containing generative AI contributions from work produced without them. Van Vlasselaer et al. (2026) found that accuracy depended on the detection tool, the generative model, and the way the text had been assembled; performance declined for some tools when texts were hybrid or AI-generated prose had been modified to resemble human writing. Pangram was more accurate than the other tools on that particular dataset, yet the authors still advised against allowing a detection result to determine a high-stakes decision on its own. Text origin and intellectual contribution are thus separate assessment questions. Neither can substitute for evidence that the student understands the work.

Swiecki et al. (2022) locate the difficulty across three interrelated parts of assessment. AI complicates the student model that specifies the attributes to be measured, the task model intended to elicit evidence of those attributes, and the evidence model used to interpret performance. An AI-supported product can no longer be read straightforwardly as an expression of competence that belongs exclusively to the student. The more useful question is not simply who wrote it, but which student competence it evidences, under what conditions, and with what degree of confidence.

REAM-AL approaches this problem by treating supported performance, independent competence, and transfer as complementary sources of evidence rather than interchangeable scores. Their full definitions and assessment implications are developed in Section 4.3.5.

Two research conversations have developed largely in parallel. Gifted-education scholarship has emphasized possible uses for writing, personalization, differentiation, inquiry, and mentoring, whereas research with broader populations has examined cognitive offloading, metacognitive accuracy, agency, ownership, assessment validity, and transfer (Almumen & Jouhar, 2025; Darvishi et al., 2024; Fan et al., 2025; Guilbault et al., 2025; Kofinas et al., 2025; Urban et al., 2025).

What remains missing is a convincing account of how these strands meet in the development of advanced children. The literature reviewed here yielded no model specific to advanced learners that could address three questions together: how far AI-supported advanced performance represents independent competence; how the system’s functional contribution can be separated from the student’s epistemic decision authority; and how intellectual ownership connects with competence that can be demonstrated independently and transferred elsewhere. A developmental framework is needed precisely because the immediate value of AI support and its longer-term cognitive or epistemic consequences may not be the same.

Against this background, the study asks whether thinking with generative AI merely makes advanced children’s existing capacities more visible and usable or gives rise to a relational form of performance in which cognitive work, epistemic decisions, and intellectual ownership are redistributed between child and technology. “Relational” does not imply that generative AI creates a new, independent form of human intelligence. It describes a change in the conditions under which advanced performance is produced, supported, observed, assessed, and ultimately attributed to the student.

The aim is to build a theoretical account of how advanced learners think with generative AI. Developmental and contextual theories of giftedness are brought into conversation with work on extended and distributed cognition, cognitive offloading, metacognitive development, epistemic agency, intellectual ownership, independent transfer, and assessment validity. The resulting framework is the Relational Epistemic Agency Model for Advanced Learners (REAM-AL).

REAM-AL organizes five focal constructs across four analytical layers. Developmental epistemic readiness describes a learner condition; cognitive task distribution and epistemic decision distribution describe interaction processes; intellectual ownership concerns the learner’s epistemic relation to the resulting work; and the supported performance–independent competence–transfer relationship provides the evidentiary basis for developmental interpretation. Relational cognitive extension and developmental substitution are not additional components or fixed learner types. They are provisional interaction patterns inferred from converging process and performance evidence.

## 2. Theoretical and Conceptual Framework

### 2.1. Intelligence and Giftedness: From Individual Capacity to Relational and Developmental Performance

Defining giftedness is not simply a theoretical exercise. The definition adopted influences which students are identified, what forms of educational provision they can access, and how their performance is understood. Psychometric measures remain valuable for identifying cognitive differences among learners. A test score obtained at a single point in time, however, says little about the conditions under which potential will—or will not—develop into advanced performance. Contemporary talent-development theories retain cognitive capacity as a central consideration while placing the developmental path from potential to accomplishment firmly within its educational and social context.

This developmental emphasis is evident in Gagné’s Differentiated Model of Giftedness and Talent, which separates natural abilities from the domain-specific competencies acquired through systematic learning and practice. Their transformation depends on more than instruction alone. Personal and environmental catalysts may accelerate, redirect, or constrain the process (Gagné, 2004, 2015). Subotnik et al. (2011) describe a related trajectory from potential through competence and expertise to creative productivity in some domains. What matters along that path changes over time: domain demands, high-quality learning opportunities, and psychosocial skills do not carry the same weight at every stage. Olszewski-Kubilius et al. (2015) similarly emphasize the joint development of cognitive and psychosocial resources, with the balance between them shifting as talent develops. High cognitive capacity, on this account, is not the culmination of development. It is one of its starting conditions.

A relational view shifts the question from what a learner possesses to what becomes possible within a particular system. In the Actiotope Model, development arises from the interaction between students’ internal learning resources and the external educational resources available in their environment (Ziegler et al., 2017). Barab and Plucker (2002) make a comparable argument, describing ability not as a fixed property housed within the individual but as a functional relationship between the person and the physical and sociocultural world. Competent performance takes shape through this relationship. Cognitive characteristics matter, but so do task structure, available tools, social relations, and opportunities to participate. The performance observed in one setting cannot be treated as a transparent or context-free expression of individual capacity.

Even the research categories used to describe giftedness are less stable than they sometimes appear. Popp et al. (2025), in a second-order scoping review of 23 meta-analyses published between 2000 and 2023, identified inconsistent definitions and operational criteria for giftedness and talent as a major methodological concern. Two groups labeled “gifted” in different studies may have been selected according to substantially different criteria. Comparisons across studies must be read with those differences in view.

Questions of identification make the contextual problem concrete. Peters et al. (2019) found that some demographic groups were disproportionately represented among students identified as gifted. Siegle et al. (2026), examining three states, reported only limited alignment between identification procedures and the programs available to the students who were selected. Recognition and provision are thus intertwined: the criteria used to identify potential, and the educational opportunities that follow, can shape whose abilities become visible and whose have the chance to develop.

For this reason, advanced learner is used here as a broader, study-specific term rather than as a synonym for formally identified giftedness. It includes children and adolescents who learn more rapidly than their age peers within a particular domain, engage with concepts at unusual depth, demonstrate complex forms of reasoning, or show potential for creative production. Formal identification remains important. The broader term serves a different purpose: it keeps within the theoretical frame students whose potential may be overlooked because identification practices and educational opportunities vary across contexts.

Generative AI adds a further layer to the already unsettled relationship between capacity and performance. Once the system contributes information, ideas, organization, or prose, a polished product no longer provides a straightforward measure of what the student can do independently. The observed performance may combine the learner’s domain knowledge, intentions, choices, and oversight with content and operations supplied by AI. The product remains informative, but the inference that can be drawn from it is narrower than its quality may initially suggest.

Direct empirical work with gifted students is still sparse. Almumen and Jouhar (2025) studied five gifted university seniors and found that using ChatGPT 3.5 for prewriting brainstorming was associated with higher persuasive-writing scores. Hasanbaşoğlu and Baloğlu (2026) followed nine academically gifted first-year university students who used ChatGPT while planning, drafting, and revising academic work. The students valued its efficiency, adaptability, and potential for indirect learning, yet also described risks involving academic integrity, reliability, originality, and dependence. These small university samples cannot stand in for advanced children and adolescents. They do, however, expose the inadequacy of treating generative AI as uniformly beneficial or harmful.

The more revealing question lies beneath that binary judgment. How is cognitive work divided between learner and system, and what happens to the learner’s independent development as that division changes? Product quality can tell only part of that story.

### 2.2. Human–AI Co-Thinking: Extended and Distributed Cognition

The use of external resources in cognitive activity long predates generative AI. Writing, diagrams, notebooks, calculators, and digital information systems have long participated in remembering, comparing, calculating, and deciding. Theories of the extended mind and distributed cognition take this observation seriously. External resources are not always tools consulted after thought has taken place; under certain conditions, they become part of how cognition itself is organized and carried out (Clark & Chalmers, 1998; Hollan et al., 2000; Hutchins, 1995).

Although closely related, the two traditions work at different levels of analysis. Extended-mind theory asks when a reliably and functionally integrated external resource can be regarded as part of an individual’s cognitive system (Clark & Chalmers, 1998). Distributed cognition moves beyond the individual as the sole unit of analysis. Its concern is how cognitive work is coordinated across people, artifacts, representations, and established practices (Hollan et al., 2000; Hutchins, 1995). This shift is useful for the present argument because it allows generative AI to be examined not merely as a tool in the student’s possession, but as a component capable of changing how a task is performed and how cognitive contributions are distributed within it.

An AI-generated response may prompt a student to reframe a problem, formulate a new question, or reconsider an earlier assumption. The student may set the goal, compose a prompt, interpret the answer, detect an error, and choose among alternatives. Meanwhile, the system contributes explanations, comparisons, examples, or possible solutions. Distributed cognition offers a language for examining how this work moves between learner and technology over the course of an interaction. Hollan et al. (2000) and Hutchins (1995), of course, were not writing about generative AI. Applying their ideas here is a conceptual extension of those theories rather than a claim already established in the original literature.

The term co-thinking requires a similar qualification. It does not place human beings and AI on the same plane of mental experience, intention, or epistemic standing. A large language model can produce fluent, context-sensitive language, but fluency does not establish that the system understands its statements as humans do, believes the claims it generates, or assumes responsibility for their truth. Describing language models with human terms such as “thinking,” “knowing,” and “believing” can invite an anthropomorphic reading of what these systems do (Bender et al., 2021; Shanahan, 2024). Co-thinking is used here in a narrower, functional sense: a cognitive task unfolds through repeated exchanges between a student’s actions and AI-generated outputs. The system’s functional contribution to that process must still be distinguished from the epistemic responsibility expected of the student.

For advanced learners, the distinction carries particular weight. A student with substantial domain knowledge may use AI to generate a counterexample, test competing explanations, or represent a complex problem in several ways. High cognitive capacity does not guarantee, however, that the exchange will develop into sustained critical inquiry. Kang and Lee (2023) examined 12 gifted middle school students in a physics activity and found that only 39 of the 762 questions they posed to ChatGPT—5.1%—were classified at a high cognitive level. The sample was small and tied to a single domain, so broad generalization would be unwarranted. Its more limited lesson is still important: giftedness alone did not turn access to AI into higher-order questioning.

The same learner may use AI to challenge an argument and restructure a problem in one task, then surrender most of the problem framing, evidence selection, and reasoning to the system in another. Both interactions can yield polished work, but they do not involve the same intellectual contribution or necessarily offer the same developmental return. Evidence that product gains and later learning can diverge is examined in Section 2.3 and Section 2.6 (Fan et al., 2025). Successful completion by the combined human–AI system cannot, on that basis alone, establish that the student has learned the relevant cognitive operations.

Two questions must consequently be kept apart. How is cognitive work distributed between human and technology? And what does that distribution mean for the learner’s development? This distinction provides the theoretical basis for REAM-AL’s separation of interactions that extend a student’s intellectual possibilities from those in which AI takes the place of processes the student is expected to develop.

### 2.3. Cognitive Offloading: The Boundary Between Strategic Support and Developmental Substitution

Cognitive offloading refers to the use of physical actions or external resources to reduce or reorganize the information-processing demands of a task (Risko & Gilbert, 2016). Writing something down, setting a reminder, assigning a calculation to a device, and searching online for needed information are familiar examples. None removes cognitive effort altogether. Instead, they alter how the operations involved in the task are divided between the person and the external resource. Even the expectation of later access can influence what people choose to remember. Sparrow et al. (2011), for instance, found that participants were more likely to remember where accessible information could be found than the information itself.

Generative AI greatly expands the range of operations that can be offloaded. Traditional external-memory tools mainly store and retrieve information recorded earlier; generative systems can produce options, arguments, prose, and possible solutions (Seung & Basham, 2026). Yet these uses are not cognitively equivalent. A student who asks for a counterexample, invites the system to locate weaknesses in a solution, or uses an alternative perspective to test an argument may delegate part of the work while retaining control over its direction and standards of judgment. By contrast, requesting a solution before making sense of the problem—or accepting generated reasoning without examining it—may shift the central work of learning to the technology. The distinction between these patterns is theoretical. It is not borrowed from an existing classification, but developed by extending the cognitive-offloading literature to generative AI.

How offloading affects development cannot be inferred from the amount of AI use alone. The position of the delegated process within the learning goal matters, as does the degree of control the student retains. Offloading can improve performance during a task while weakening later memory for delegated information and, in some circumstances, limiting skill acquisition (Grinschgl & Neubauer, 2022). Research on generative AI points to a similar separation between immediate performance and subsequent learning. Product gains have not always been accompanied by comparable gains in knowledge, transfer, or delayed retention (Barcaui, 2025; Fan et al., 2025). These findings make product quality an insufficient proxy for durable learning, while the full implications for advanced children remain to be tested.

Seung and Basham (2026) use strategic cognitive offloading to distinguish external support that facilitates learning from the excessive delegation of core cognitive processes. For an advanced learner, strategic use might involve accelerating routine language correction, generating alternatives to an existing solution, or sorting a large body of sources at an early stage. The crucial issue is what the student continues to do. Can the learner question the system’s suggestions, reject weak options, explain the basis for a choice, and present the central solution without generative support? Evidence of those capacities would make it more plausible that offloading has created room for thought rather than replaced it.

REAM-AL uses developmental substitution for a different arrangement: generative AI assumes cognitive or epistemic operations that the learner is expected to perform as part of the learning goal. The concept does not come from a pre-existing scale. It emerges from reading research on the possible learning costs of cognitive offloading alongside evidence that supported products, knowledge acquisition, and transfer may follow different trajectories (Fan et al., 2025; Grinschgl & Neubauer, 2022; Seung & Basham, 2026). When problem framing or evidence evaluation lies at the center of a task, repeatedly assigning that work to the system may improve the product. It does not establish that the student has developed the same competence independently.

Developmental substitution may be particularly difficult to detect in advanced learners. Strong domain knowledge, verbal fluency, and a keen eye for patterns can enable a student to assemble AI-generated components into a coherent and persuasive whole. The finished work may appear well beyond age expectations even though the student contributed little to framing the problem, evaluating the evidence, or constructing the reasoning. Within REAM-AL, the possibility that product quality can conceal the distance between independent competence and system contribution remains a proposition to be tested empirically. Early studies with gifted university students indicate that ChatGPT can support writing and idea generation, but small samples and the absence of independent-transfer measures leave the developmental implications unresolved (Almumen & Jouhar, 2025; Hasanbaşoğlu & Baloğlu, 2026).

### 2.4. Metacognitive Development and Developmental Epistemic Readiness

Metacognition encompasses both what individuals know about their own cognitive processes and their ability to monitor and regulate those processes (Flavell, 1979; Nelson & Narens, 1990). Knowing a strategy, however, is not the same as knowing when or how to use it. Monitoring and control continue to develop throughout childhood, and the two do not always progress in step. Bayard et al. (2021) documented age-related improvements in both capacities, alongside difficulties that persisted over time. O’Leary and Sloutsky (2017) further showed that feedback and strategy instruction may affect different components of metacognition: feedback can sharpen performance monitoring, whereas strategy instruction may alter control decisions. Strategy knowledge, accurate self-evaluation, and the ability to choose an appropriate course of action should not be treated as a single competence.

Limited evidence indicates that gifted students can use sophisticated problem-solving strategies in some settings. García-Moya et al. (2024), for example, followed a gifted child aged 10 years and 7 months through a series of non-routine mathematical problems. The student searched for patterns, set intermediate goals, used one-to-one correspondence, and worked through trial and error. Yet this was not an unsupported display of advanced metacognitive control. The case involved 30 individual sessions structured around Pólya’s problem-solving stages, and the student required assistance from the researcher during some activities. One guided case cannot establish that high cognitive capacity brings equally advanced metacognitive control across domains and tasks.

Generative AI could be designed to support such development. It might prompt students to explain their reasoning, compare alternative solutions, or respond to questions that guide self-evaluation. Merely providing access to the technology will not produce these effects; the interaction must be structured so that metacognitive activity is actually elicited. In a four-week quasi-experimental study with 68 university students, Xu et al. (2025) found that explicit metacognitive support improved self-regulated learning, particularly task strategies and self-evaluation, without producing a significant between-group difference in academic achievement. Liu et al. (2026) approached the issue with elementary school students, comparing adaptive and pre-planned metacognitive scaffolds in generative AI-supported programming. Their work draws attention not only to whether support is present, but also to when it is offered and how closely it responds to the learner’s needs. The two studies involve different ages and tasks, yet together they make a persuasive case for treating timing and adaptation as part of the support itself.

Evaluating AI-generated information introduces a second metacognitive demand: students must judge not only their own performance but also the reliability of what the system provides. Urban et al. (2025) explored this problem with 98 university students. ChatGPT support increased the inclusion of accurate information in written work, but it did not significantly reduce the use of false information generated by the system. Greater reliance on inaccurate content was associated with metacognitive bias and with students’ epistemic beliefs. The participants were university students, so the result cannot be carried directly into advanced-child populations. Its relevance lies elsewhere. AI may make correct information easier to use without making unreliable information easier to recognize.

REAM-AL introduces developmental epistemic readiness to capture a learner’s current capacity to understand and question an AI-generated claim, compare it with other evidence, recognize uncertainty, and justify a decision to accept or reject it. The construct brings together work on children’s metacognitive development, the evaluation of AI output, and the effects of explicit metacognitive support (O’Leary & Sloutsky, 2017; Urban et al., 2025; Xu et al., 2025). It is not a validated scale or an established student attribute. At this stage, it is a developmental proposition that requires direct examination in advanced-learner samples.

Crucially, developmental epistemic readiness is not another name for general intelligence. An advanced learner with strong domain knowledge may quickly detect an obvious factual error and still struggle with a false argument presented in fluent, persuasive language. The ability to write elaborate prompts creates a similar risk of overinterpretation. It demonstrates a form of interactional skill, but not necessarily the capacity to decide which evidence is stronger and why. These are not empirically established profiles of advanced learners. They are theoretical possibilities that explain why cognitive capacity and the evaluation of AI-generated claims should remain analytically distinct. Educational support should be calibrated accordingly to the learner’s age, domain knowledge, metacognitive control, and the learning goals of the task.

Metacognitive control tells us how students monitor and regulate their own thinking. It cannot tell us, on its own, who made the central knowledge-related decisions during an interaction. That question requires a separate examination of epistemic agency and decision authority.

### 2.5. Epistemic Agency and the Distribution of Decision-Making Between Humans and AI

Epistemic agency involves more than completing an assigned task. It becomes visible when students help determine which questions are worth pursuing, develop and evaluate ideas, shape the goals of inquiry, and justify the conclusions they reach. In the knowledge-building literature, students exercise epistemic agency by participating purposefully in collective knowledge creation, assuming responsibility for epistemic goals, and influencing the direction in which ideas develop (Damşa et al., 2010; Scardamalia, 2002). Visibility is not the decisive criterion. A learner may appear highly active while having little influence over the standards and direction of knowledge production.

This distinction becomes easy to miss in AI-supported work. A student may produce dozens of prompts and extensively revise the generated text, yet accept the system’s problem framing and evidence without question. Another student may interact with the system only briefly but compare its suggestions against personally defined criteria, verify claims through independent sources, and reject proposals that do not withstand scrutiny. The amount of interaction tells us little about epistemic agency on its own. REAM-AL is concerned instead with where authority over the problem, method, evidence, and conclusion resides during the exchange.

Advanced learners may be particularly adept at composing elaborate prompts because of their domain knowledge, verbal fluency, or technical skill. Prompting competence is not, however, equivalent to epistemic authority. The former is an instrumental capacity: it enables a learner to initiate and steer an exchange with the system. Epistemic authority concerns a different set of decisions—what problem should be investigated, which method is appropriate, what counts as sufficient evidence, and which conclusion can reasonably be defended. This is a conceptual distinction rather than an empirically validated classification of advanced learners. Its purpose is to prevent technical fluency from being mistaken for authority over knowledge production.

The system’s functional contribution must also be kept separate from the student’s epistemic authority. AI may retrieve information, generate alternatives, compare options, provide examples, or revise prose. Such contributions can be extensive. Their extent neither proves that decision authority has shifted to the system nor confirms that the student has retained it. Distributed cognition helps explain how cognitive work is organized across people and tools (Hollan et al., 2000); epistemic agency directs attention to the learner’s responsibility for knowledge goals and the development of ideas (Damşa et al., 2010; Scardamalia, 2002). REAM-AL brings these two perspectives into the same analytical frame while preserving the difference between doing work within a task and holding authority over its epistemic direction.

Within that frame, the distribution of epistemic decision-making refers to how authority over problem framing, method selection, evidence evaluation, the central claim, and the final conclusion is allocated among the student, generative AI, and, where relevant, the teacher. It is not a validated scale or an established classification system. Rather, it is an analytical construct developed to examine cognitive distribution and epistemic responsibility together. Counting prompts or measuring time spent with AI would miss its central concern: the decision architecture through which knowledge is produced.

That architecture may shift repeatedly within a single task. A student might define the initial problem independently, accept AI-generated evidence with little scrutiny in the middle of the process, and later recover an active role when drawing the conclusion. Epistemic authority should not be assumed to remain constant simply because the same learner is involved throughout. Problem framing, source selection, evidence evaluation, claim construction, and conclusion drawing need to be examined separately if we are to see where the student retains authority and where it moves elsewhere. This process-oriented account has not yet been tested with advanced learners. It remains one of REAM-AL’s empirical propositions.

### 2.6. Intellectual Ownership, Independent Transfer, and Assessment Validity

An AI-supported product may emerge from a complicated mixture of the student’s prior knowledge, prompts, choices, and revisions and the content supplied by the system. The finished work rarely shows how those contributions were divided. A student who wrote relatively little of the text may still have framed the problem, established the central claim, and selected the evidence according to personally defined criteria. Another may rewrite many of the system’s sentences while leaving its problem framing and intellectual structure largely intact. Word counts and visible edits cannot, by themselves, tell us who did the consequential thinking.

Intellectual ownership is used here in an epistemic rather than a legal sense. It refers to whether students understand the product’s central intellectual structure, can distinguish their contribution from that of AI, and are able to defend the position they have adopted with reasons. Ebrahimzadeh et al. (2026) regard the presentation of AI-generated content that a student does not understand as a threat to coauthorship integrity and assessment validity. Their response is to orient assessment toward verifying the student’s epistemic ownership of the writing. REAM-AL develops intellectual ownership from this position as a theoretical construct, not as a validated psychometric attribute. Nor is ownership equivalent to disclosure. A declaration of AI use provides information about transparency and academic integrity; it does not establish that the student understands, can justify, or is able to defend the work.

This distinction reaches directly into assessment validity. Validity is not a permanent property of a test or score. It concerns the extent to which interpretations of student performance—and the uses made of those interpretations—are supported by appropriate evidence (American Educational Research Association [AERA] et al., 2014). Once AI contributes content, structure, or reasoning to an assessed product, the competence represented by the observed performance becomes less clear. If the combined system’s performance is attributed directly to the student without additional evidence that the student carried out the central intellectual operations, the alignment between the intended construct and the observed performance may be weakened (Swiecki et al., 2022).

Detection does not resolve this validity problem. Kofinas et al. (2025) found that assessors could not reliably distinguish among human-written texts, AI-modified texts, and texts generated entirely by AI. Van Vlasselaer et al. (2026) likewise reported that detection performance varied with the tool, the generative system, and the way a text had been produced; they cautioned against using such tools as the sole basis for high-stakes decisions. More fundamentally, even accurate identification of AI involvement would not show whether the student understood the product or could demonstrate the relevant competence in a new task. “Who produced this text?” is only one assessment question. A more consequential one is what defensible evidence the product provides about the student’s competence and how far that interpretation can be sustained (American Educational Research Association [AERA] et al., 2014; Swiecki et al., 2022).

Evidence collected beyond the supported task can clarify what remains attributable to the learner. Fan et al. (2025) observed greater improvement in the written products of university students using ChatGPT, but no significant advantage in knowledge acquisition or transfer over other support conditions. Wu et al. (2025), across four online experiments involving 3562 adults, found that human–generative AI collaboration improved performance during the task, yet those gains did not generalize consistently to subsequent independent work. Neither investigation involved advanced children, so neither provides direct population-specific evidence. Their value is mechanistic: both show why supported product performance should be distinguished from competence demonstrated when AI does not perform the target operation, and why transfer to a different task must be examined separately.

These findings motivate distinctions formalized in Section 4.3.5. REAM-AL does not treat supported performance, independent performance, and transfer as three ordered levels. Instead, it separates the availability of AI support, whether AI performs the target operation, the novelty of the task, and the timing of assessment. Interaction records, prompt histories, product defenses, delayed tasks, and carefully specified follow-up conditions can then provide complementary evidence about participation in the human–AI system, competence attributable to the learner, and the use of knowledge or strategies beyond the original task.

### 2.7. Theoretical Synthesis: Why Is REAM-AL Needed?

Talent development, distributed cognition, cognitive offloading, metacognition, and epistemic agency each illuminate a different aspect of AI-supported performance. None, however, is sufficient on its own to explain the relationship between the success of the combined human–AI system and the student’s development. Cognitive work may be distributed efficiently between learner and technology without the learner acquiring the processes carried out by the system. Likewise, students may monitor their own performance carefully while surrendering authority over the problem, the evidence, or the direction of the inquiry.

Across these studies, the recurring tension is that stronger performance during AI use may not be accompanied by comparable gains in knowledge, delayed retention, or subsequent independent performance (Barcaui, 2025; Fan et al., 2025; Wu et al., 2025).

REAM-AL was developed to provide that account. It brings into a single analytical frame the learner’s readiness to evaluate AI output, the distribution of cognitive work and epistemic decisions, intellectual ownership of the product, and competence demonstrated when AI does not perform the assessed target operation. The model looks beyond the quality of the final product to distinguish interactions that extend a learner’s thinking from those that take the place of processes the learner is expected to develop. These components are not validated classifications. They are theoretical propositions intended for empirical examination with advanced learners.

## 3. Method

### 3.1. Research Design

This research was designed to develop a theoretical model of how advanced children and adolescents think with generative AI. Sources were identified through a structured database search and examined using an integrative literature review. This design allows evidence drawn from different methodological traditions and theoretical perspectives to be compared around a shared problem (Snyder, 2019; Torraco, 2016; Whittemore & Knafl, 2005).

This review brought together literature on giftedness and talent development, extended and distributed cognition, cognitive offloading, metacognitive development, epistemic agency, intellectual ownership, independent transfer, and assessment validity. The intention was not to summarize these fields one after another, but to trace explanatory connections among them and use those connections to construct an empirically testable model (Jaakkola, 2020). The research is neither a systematic review nor a meta-analysis, and it does not claim exhaustive coverage of every relevant publication. Search, deduplication, source selection, and claim–evidence mapping procedures were nevertheless documented to make the development of the model more transparent and traceable.

### 3.2. Scope of the Review and Guiding Questions

The review was organized around a central problem: Under what conditions does an advanced product created with generative AI reflect relational performance that extends a student’s thinking and development, and under what conditions does it reflect the transfer of cognitive and epistemic processes the student is expected to develop to the technology?

Three questions guided the analysis:

1. How do relational, developmental, and contextual approaches to human intelligence and giftedness inform the interpretation of advanced performance supported by generative AI?
2. How can the distribution of cognitive and epistemic work between the student and generative AI be explained through extended and distributed cognition, cognitive offloading, metacognitive development, and epistemic agency?
3. What roles do intellectual ownership, independent transfer, and assessment validity play in evaluating the relationship between an AI-supported product and the student’s independent competence?

The analysis was not organized around a general judgment that AI is either beneficial or harmful. Alongside product quality and task success, attention was given to those who framed the problem, evaluated the evidence, and selected the central claim; how well the student understood and could defend the product; and whether the relevant intellectual processes could be sustained once generative support was reduced.

### 3.3. Information Sources and Search Process

Structured database searches were conducted on 24–25 August 2026. The Scopus search was completed on 24 August, followed by ERIC on 25 August. Given the scope of the research problem, the search was deliberately interdisciplinary, spanning education, psychology, gifted education, human–computer interaction, educational assessment, and AI ethics.

The main record pool consisted of results exported from Scopus and ERIC. Twelve conceptual search clusters were used in Scopus to capture different dimensions of the research problem. These covered generative AI and advanced learners; metacognition and epistemic agency; assessment validity; intellectual ownership and human–AI contributions; independent performance and transfer; creativity and co-creation; inequalities in access to AI; and cognitive and epistemic sustainability. The ERIC search used three broader clusters focused on the direct intersection of generative AI and advanced learners, cognitive and epistemic processes, and AI-supported assessment and educational equity. Because a single publication could appear in more than one cluster, the raw results were treated as exported record entries rather than as counts of independent studies.

**Figure 1:**
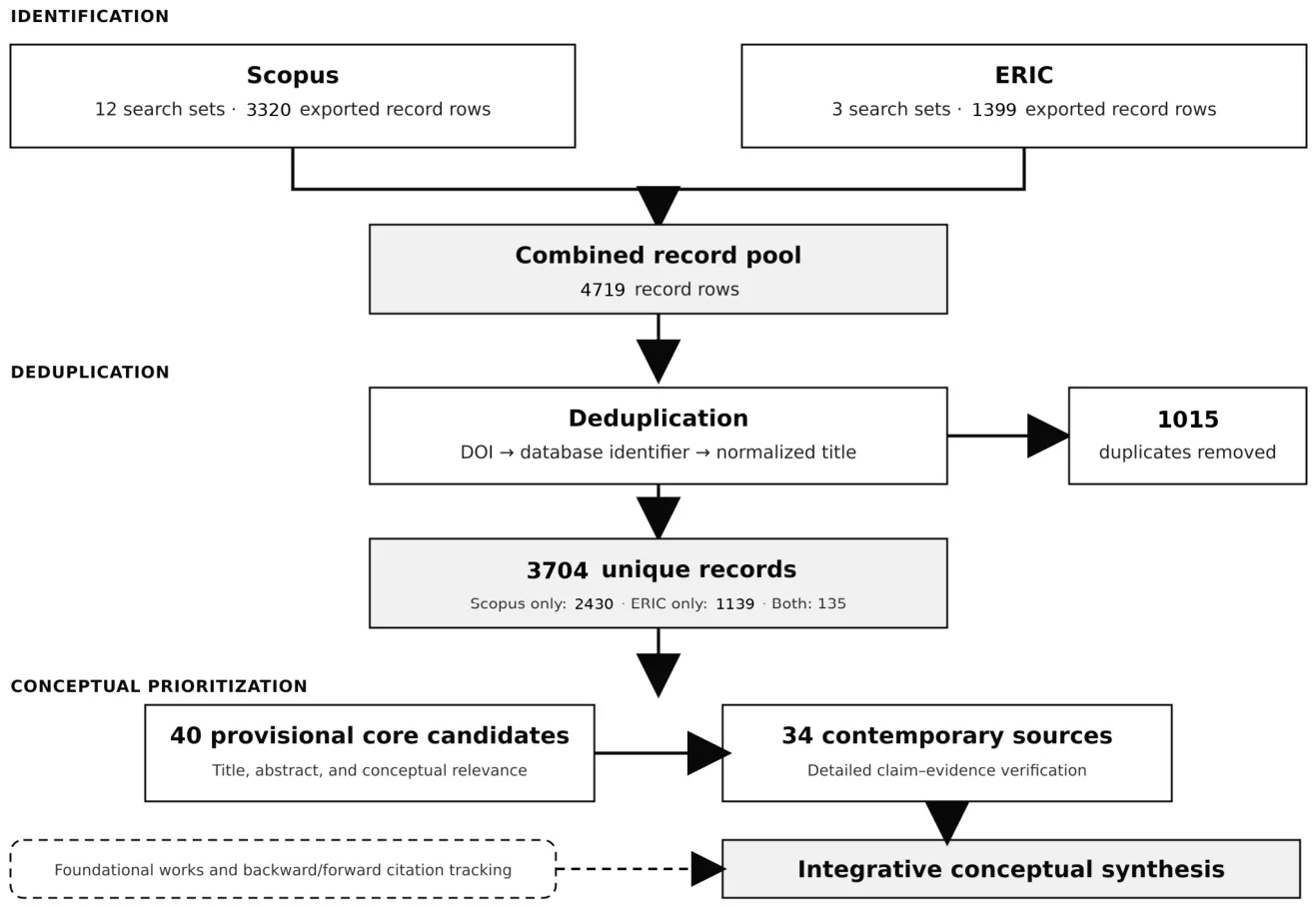
Database record generation and conceptual prioritization processes. Note. The preliminary identification of core candidates does not imply that all other unique records were definitively excluded. The 34 sources shown in the figure are current publications that underwent detailed claim–evidence verification; they do not represent the total number of sources included in the synthesis. Foundational theoretical works and additional publications identified through backward and forward citation searching were also incorporated into the integrative synthesis.

Figure 1 presents the process through which database records were combined, deduplicated, and conceptually prioritized.

### 3.4. Search Strategy

Search terms were organized into conceptual clusters derived from the guiding questions and adapted to the syntax of each database. The main clusters covered generative AI and human–AI collaboration; giftedness, high ability, and advanced learners; extended and distributed cognition and cognitive offloading; metacognition, self-regulation, and epistemic agency; intellectual or epistemic ownership and human contribution; knowledge retention, independent performance, transfer, and assessment validity; creativity and co-creation; and digital inequality and cognitive and epistemic sustainability. Synonymous and closely related terms within a cluster were combined with OR, while AND was used to connect different clusters.

Broad queries were chosen to maximize search sensitivity and accommodate the varied terminology used to describe advanced learners. Some retrieved records were therefore expected to fall outside the final conceptual scope, and appearance in the search results did not lead automatically to inclusion in the synthesis. In the first Scopus cluster, 286 records were displayed before the 2022–2026 publication-year filter was applied, and 113 records were exported after that filter. Supplementary Table S1 reports the database, search date, search cluster, available query string, filters, displayed results, and exported records. Complete query strings were preserved for nine of the twelve Scopus clusters. The archived search log did not retain the exact strings for three Scopus clusters (E, E1, and E2) or the three ERIC clusters; the applied ERIC filters and pre-export retrieval counts were also not retained. These entries are identified explicitly in the supplement rather than reconstructed retrospectively. The corresponding exported files and record counts were retained and verified. Because the missing information cannot be recovered from the archived records, its effect on search coverage cannot be determined.

Because research at the direct intersection of generative AI and advanced learners remains limited, the search was not confined to queries combining population and technology terms. Separate mechanism-oriented searches were conducted for the model’s core components. A study of metacognitive development in children or adolescents, for example, could be treated as adjacent-population evidence when it helped explain developmental epistemic readiness, even if giftedness was not its primary focus. Studies involving university students or adults were used as mechanism evidence when they directly compared supported product quality with knowledge acquisition, retention, or transfer. Such results were not generalized to advanced children and adolescents. They served instead as indirect evidence for relationships that REAM-AL proposes for future testing.

For research specifically concerned with generative AI, priority was given to publications from 2022 to 2026. No date restriction was applied to the theoretical foundations of intelligence and giftedness, extended and distributed cognition, cognitive offloading, metacognition, epistemic agency, or assessment validity. Earlier work was retained when it provided a foundational account of a concept or continued to serve as a central point of reference in contemporary research.

Database searching was supplemented by backward reference searching in the bibliographies of priority publications and forward citation tracking of relevant studies. Peer-reviewed research formed the main evidence base, although official documents issued by international bodies and professional organizations were consulted where the research problem required them. These documents did not substitute for empirical evidence. They were used as normative or contextual sources when discussing principles of ethical human oversight, AI competencies, and assessment.

### 3.5. Eligibility Criteria and Source Prioritization

Candidate records were assessed on the basis of their titles, abstracts, keywords, publication types, samples, research designs, and relevance to the explanatory problem addressed by REAM-AL. To proceed to detailed review, a source needed to contribute to at least one of the following areas: talent development; metacognitive or epistemic processes; the distribution of cognitive work and decision-making in human–AI interaction; the effects of generative AI on learning or creativity; relationships among supported performance, retention, and transfer; or intellectual ownership and assessment validity. Systematic reviews, scoping reviews, meta-analyses, and formal scientific or professional frameworks were also considered when they contributed directly to the research problem.

Given the limited number of studies conducted directly with advanced learners, evidence from adjacent populations, mechanism-focused research, and contextual studies was also examined. Lower priority was assigned to publications that reported only technical system performance without an educational or cognitive connection, lacked a verifiable method or publication source, duplicated another record, or made no meaningful contribution to the model’s explanatory problem. Preprints and opinion pieces were used only as supplementary sources when they brought a current theoretical debate into view, and no peer-reviewed equivalent was available. Core empirical claims were not based on these materials.

During the initial title-and-abstract screening, records were assigned to one of four provisional priority levels according to their conceptual proximity to the research problem. The classification presented in Table 1 did not constitute a final inclusion or exclusion decision. It served as a screening device for determining the order in which records would receive detailed review.

The 40 studies initially identified as core candidates underwent human review. Their DOIs and publisher records, research designs, samples, and reported results were checked again. Of these, 34 proceeded to detailed claim–evidence assessment. This number refers to the current high-priority sources selected for in-depth verification; it does not represent either the complete body of sources used in the article or a closed research sample.

### 3.6. Evidence Layers

Conceptual proximity helped determine the order in which sources were reviewed, but it could not establish how directly their findings applied to advanced children and adolescents. Sources selected for detailed review were therefore classified into five evidence-proximity categories based on population and inferential role. Table 2 separates direct evidence from advanced children and adolescents, evidence from gifted or advanced university students, general child and adolescent evidence, university or adult mechanism evidence, and theoretical extrapolation. The categories do not rank methodological quality; they show how far a claim travels before it reaches the target population.

### 3.7. Data Extraction and Conceptual Analysis

A standardized data-extraction procedure was applied to every source selected for detailed review. The recorded fields included bibliographic information and DOI, where available; publication type and research design; sample or theoretical context; principal finding or theoretical contribution; the relevant REAM-AL component; evidence layer; intended use within the article; and limitations on applying the source to advanced children and adolescents. This structure made it possible to trace both the function of each source in the manuscript and the claims it was used to support.

Conceptual analysis proceeded iteratively. Rather than summarizing one discipline after another, the analysis examined how the different bodies of literature addressed the shared research problem. Building a conceptual framework from multidisciplinary scholarship requires concepts to be defined and compared, relationships among them to be established, and the resulting ideas to be reorganized into an explanatory whole (Jabareen, 2009). That approach was combined here with the logic of theory synthesis and conceptual model development (Jaakkola, 2020). The analysis comprised five stages.

#### 3.7.1. Mapping the Theoretical Domains

The first stage mapped the literature on relational and developmental approaches to giftedness, extended and distributed cognition, cognitive offloading, metacognitive development, epistemic agency, intellectual ownership, human–AI contributions, independent transfer, assessment validity, creativity, and co-creation. These domains were not treated as a sequence of self-contained topics. Each was approached as a body of knowledge capable of explaining AI-supported performance among advanced learners at a different level of analysis.

#### 3.7.2. Comparing Concepts and Establishing Their Boundaries

Next, the core concepts were compared in terms of their definitions, assumptions, units of analysis, and explanatory limits. Extended and distributed cognition, for example, explain how cognitive work is organized across people, tools, and representations, but they do not establish what the student learns from that arrangement. Metacognitive control concerns how students monitor and regulate their own thinking. Epistemic agency addresses their decision-making position in relation to the problem, evidence, method, and conclusion. Drawing these boundaries was necessary to prevent conceptually adjacent terms from being used interchangeably and to specify the role each would play within the model.

#### 3.7.3. Identifying Theoretical Tensions and Explanatory Gaps

The sources were also read for disagreement, divergence, and unresolved tension, not only for mutually reinforcing conclusions. Several distinctions were maintained throughout the analysis: supported product quality versus independent competence; cognitive extension versus developmental substitution; AI’s functional contribution versus the student’s epistemic decision authority; and disclosure of AI use versus intellectual ownership. Short-term task success was considered separately from knowledge acquisition, delayed retention, and transfer. Likewise, the performance of the combined human–AI system was not assumed to represent competence attributable to the student alone. In the creativity literature, attention was also given to a possible divergence between the creativity of individual products and the overall diversity of ideas generated.

These comparisons exposed a common limitation in the existing approaches: none could bring the tensions together within a developmental account of advanced children and adolescents. REAM-AL grew out of the need for an analytical structure capable of preserving these distinctions while explaining how they relate to one another.

#### 3.7.4. Constructing the Model Components

Explanatory patterns recurring across the different bodies of literature were grouped into higher-order components on the basis of conceptual proximity. Not every concept identified in the review was transferred directly into the model. A construct was retained as a distinct component only if it helped explain developmental differences in human–AI interaction, made visible a development or assessment problem specific to advanced learners, could be linked to other components on defensible theoretical grounds, and was potentially open to future investigation. Relevant forms of evidence might include observation, interviews, interaction records, think-aloud protocols, product defense, or performance tasks.

The synthesis yielded five focal constructs organized across four analytical layers rather than five equivalent or sequential components. Developmental epistemic readiness represents a learner condition. Cognitive task distribution and epistemic decision distribution describe interaction processes. Intellectual ownership represents the learner’s epistemic relation to the resulting work. The relationship among supported performance, independent competence, and transfer forms an evidentiary layer for judging what the observed performance can support an inference about. Relational cognitive extension and developmental substitution were positioned as provisional interaction patterns inferred from the configuration of these constructs, not as direct measures of benefit or harm. Longer-term changes in independent competence, transfer, self-regulation, and epistemic responsibility remain developmental outcomes to be tested longitudinally. None of these constructs or patterns is presented as an established psychometric measure.

#### 3.7.5. Developing Relationships and Testable Propositions

At the final stage, the components were not arranged as a fixed, linear sequence of development. Their proposed relationships were situated within the interaction among the learner’s developmental characteristics and domain knowledge, the functions of the AI system, the learning purpose of the task, instructional support, and assessment conditions. Each relationship was examined for its theoretical basis, the evidence layer from which it drew support, and its potential to be investigated through observation, process data, or performance measures. Relationships were not retained as model propositions when they lacked a defensible theoretical rationale, could not be linked to an identifiable form of evidence, or could not be expressed in a form suitable for empirical testing.

Relationships not yet examined directly in advanced children were presented as theoretical propositions or testable explanatory possibilities rather than as empirical findings. This distinction prevented conclusions reported directly in the source literature from being placed on the same evidential footing as concepts and relationships generated through the present synthesis.

### 3.8. Analytical Coherence, Transparency, and Limitations

Because the article was written by a single author, source selection and conceptual analysis were not independently coded by a second researcher, and no inter-rater agreement coefficient was calculated. Several procedures were used to reduce the risks associated with single-reviewer analysis. Search dates, cluster labels, filters, and record counts were documented; complete query strings were reported where they had been preserved; raw export files were retained; and deduplication followed a predetermined sequence. Rule-based prioritization determined only the order of review. Final judgments were made by the researcher after examining the full text, method, sample, reported results, and alignment between claims and evidence.

The proximity of each source to the target population was considered explicitly during interpretation. Studies involving advanced children and adolescents were distinguished from research with general child and adolescent samples, adult mechanism evidence, and theoretical or normative sources. Whenever direct application to the target population was not warranted, that limitation was reported alongside the relevant claim. The synthesis was not restricted to research emphasizing the risks of generative AI. Studies reporting benefits for learning, performance, creativity, or feedback were also included.

Empirical results and model constructs derived from the literature synthesis were not presented with the same degree of certainty. Developmental epistemic readiness, the distribution of epistemic decision-making, developmental substitution, and cognitive and epistemic sustainability were defined as theoretical propositions for future testing rather than as validated measures. An audit table was also prepared for the current sources examined in detail. For each source, it records the publication type, sample, principal contribution, relevant model component, evidence layer, and limits on generalization.

The search has three important limitations. First, database coverage was confined to Scopus and ERIC. Second, the exact strings for three Scopus clusters and all three ERIC clusters were not preserved in the archived search log; the ERIC filters and pre-export retrieval counts were also unavailable, although exported records and counts remained available. This documentation gap limits exact replication of six searches, affects 2222 of the 4719 exported record entries, and leaves its effect on search coverage indeterminate. Third, source selection and model development necessarily involved the researcher’s theoretical judgment. These limitations rule out treating the review as an exhaustive systematic search or REAM-AL as a finished, empirically validated explanatory system. The model is offered as an integrative theoretical proposal to be tested and revised across tasks, developmental periods, and conditions of AI use.

**Figure 2:**
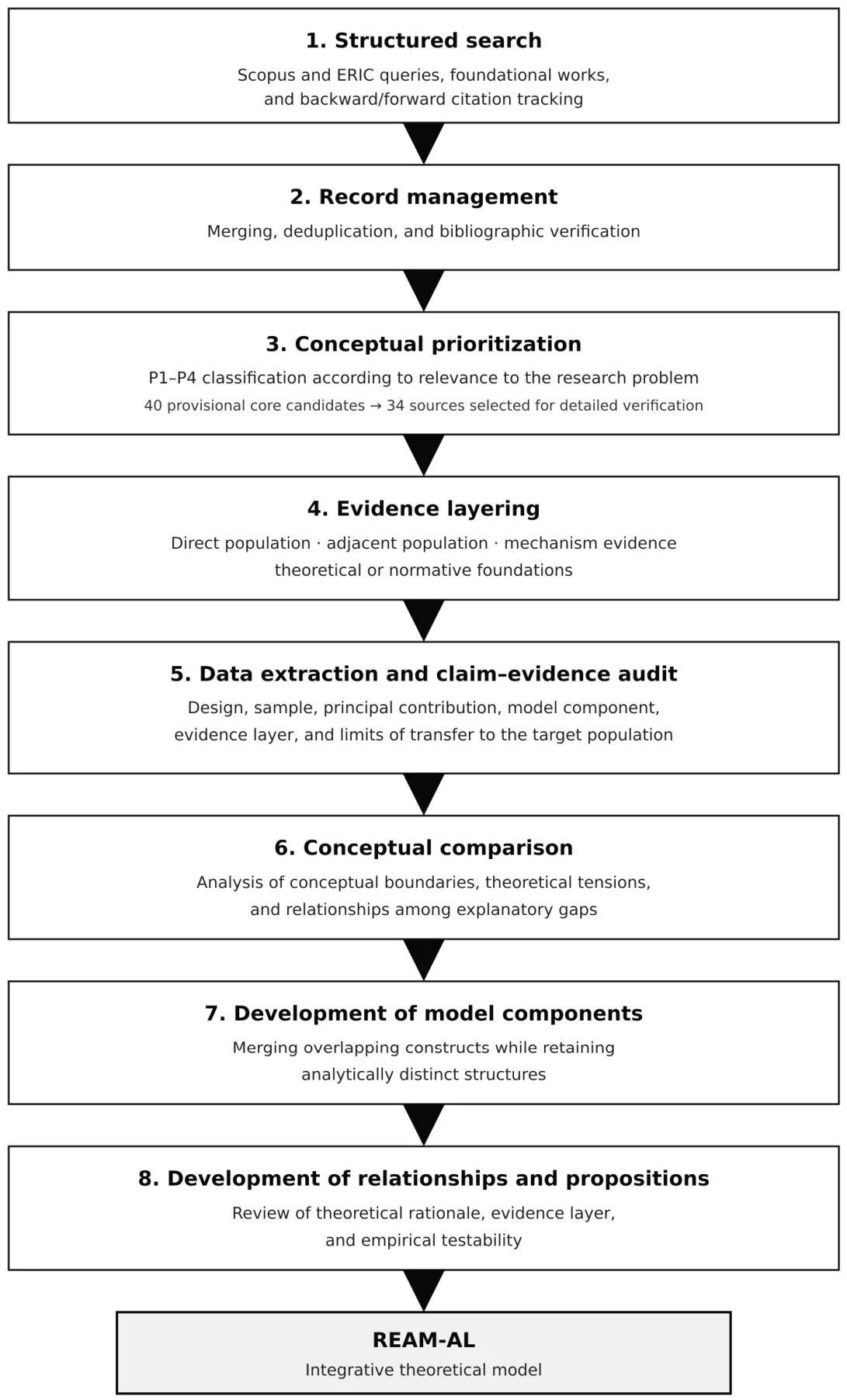
Structured search and theoretical synthesis process followed in developing REAM-AL. Note. The figure is not a PRISMA flow diagram. The 34 sources shown represent recent priority studies that underwent detailed methodological and claim–evidence verification; they do not constitute the full body of foundational and supplementary sources included in the synthesis.

Figure 2 summarizes the methodological progression from the structured search to the development of the model propositions. Unlike Figure 1, which traces the numerical flow of database records, Figure 2 shows how screening and theoretical synthesis were connected within the model-development process.

### 3.9. Ethical Considerations

No data were collected from human participants, no experimental intervention was conducted, and no personal or identifiable information was used. Because the review drew exclusively on published academic research and publicly available institutional documents, institutional ethics approval was not required. Ethical care nevertheless remained important throughout the synthesis. Sources were represented within their original contexts, the limits to generalizing across different samples were made explicit, and claims concerning advanced learners were kept within the boundaries of what the available evidence could reasonably support.

## 4. The Relational Epistemic Agency Model for Advanced Learners (REAM-AL)

REAM-AL emerged from an integrative analysis of the literature reviewed in the preceding section, spanning talent development, extended and distributed cognition, cognitive offloading, metacognitive development, epistemic agency, intellectual ownership, transfer, and assessment validity. The components, outcome patterns, and propositions presented here are not established constructs already validated under the same labels in the source literature. They are theoretical proposals developed through the present synthesis and remain to be tested in future research.

### 4.1. Purpose, Scope, and Unit of Analysis

At the heart of REAM-AL lies a straightforward question that is easily overlooked: Does an advanced product created with AI support demonstrate that the student could produce comparable work independently? The Relational Epistemic Agency Model for Advanced Learners (REAM-AL) approaches the AI-supported cognitive activity of advanced children and adolescents through this question. Two products of similar quality may arise from markedly different processes. In one case, AI may extend the student’s thinking; in another, it may perform cognitive and epistemic work that the student was expected to learn to undertake. Rather than collapsing these situations into the same appearance of success, REAM-AL seeks to make their developmental differences visible.

The term advanced learner is not restricted here to students formally identified as gifted. It encompasses children and adolescents who progress more rapidly than their peers within a particular domain, make deeper connections among concepts, display complex patterns of reasoning, or show advanced creative or academic potential. A broader designation is needed because high potential is not identified according to uniform criteria across educational systems, nor do all students have equal access to opportunities through which that potential can become visible. The absence of formal identification, then, does not necessarily mean that a learner lacks the advanced learning characteristics addressed by the model.

Nor should advanced learners be understood as a homogeneous group. Age and developmental stage, domain knowledge, previous learning experiences, metacognitive control, and access to educational opportunities may all shape what the same AI system does for different students. High cognitive capacity, moreover, offers no assurance that a learner will accurately monitor their own thinking, respond cautiously to a persuasive but erroneous output, or sustain learning without assistance. For this reason, REAM-AL does not treat cognitive potential and epistemic readiness as interchangeable.

The model is population-specific in the configuration it proposes, not because cognitive offloading, epistemic agency, or transfer occur only among advanced learners. Three features make their combination especially consequential here. First, advanced domain knowledge may enable unusually strategic use of AI while also allowing students to integrate system-generated material into products that appear independently sophisticated. Second, rapid learning, verbal fluency, and strong pattern recognition may widen the gap between the apparent level of a product and the student’s contribution to its underlying reasoning. Third, talent development depends on sustained engagement with domain-defining practices; delegating precisely those practices may therefore have different implications at different stages of domain progression. These relationships are theoretical expectations, not established population differences. Direct comparative evidence is still limited. Li et al. (2025), for example, found group differences between 197 gifted and 287 non-gifted students in reported AI-assisted scientific inquiry learning and AI literacy, but their questionnaire study does not establish the developmental mechanisms proposed by REAM-AL.

The primary unit of analysis is neither the student, the AI system, nor the final product in isolation. It is the student–AI interaction as it unfolds over time within a particular task and context. Examining that interaction requires attention not only to what was produced but also to how it came into being. Which cognitive operations did the student perform? Who made the decisions that shaped the direction of knowledge construction? To what extent can the learner take intellectual ownership of the product’s underlying structure, and which capabilities can the learner demonstrate when AI does not perform the assessed target operation? REAM-AL brings these questions together within a single interactional account.

From this perspective, fixed labels such as “dependent,” “autonomous,” or “effective AI user” are of limited value. A student may use AI to challenge an argument in one task, yet delegate nearly all problem formulation and justification to the system in another. Such positions may even shift within the same working session. What the model seeks to explain is not an enduring trait of the learner, but the particular division of cognitive and epistemic work through which a given product takes shape.

REAM-AL does not claim that the ideas it draws upon originated in this article. Its contribution is architectural: it connects constructs that have usually been discussed in separate studies and assigns each a distinct analytical role in the generative AI experiences of advanced learners. Five focal constructs are arranged across four layers: developmental epistemic readiness as a learner condition; cognitive task distribution and epistemic decision distribution as interaction processes; intellectual ownership as an epistemic relation to the resulting work; and the supported performance–independent competence–transfer relationship as an evidentiary layer. Relational cognitive extension and developmental substitution are interpretive patterns that may be inferred from how these constructs are configured in a particular task, not additional components or fixed categories of learner.

### 4.2. Core Assumptions of the Model

The first assumption challenges the idea that observed performance directly reflects the student’s individual capacity. An AI-supported product is shaped not only by the learner’s cognitive capacity and domain knowledge, but also by the structure of the task, the functions available in the system, teacher guidance, and the conditions under which performance is assessed. Because these contributions become interwoven in the final product, a high-quality output cannot establish that the student would achieve the same result independently. The product remains an important source of evidence, but the competence it represents must be examined rather than presumed.

A second assumption separates the system’s functional contribution from the epistemic authority that should remain with the learner. AI may explain a topic, generate examples, compare alternatives, or reorganize a text. Such functions can make a task easier to carry out. Epistemic authority concerns something more fundamental: Who decides how the problem will be framed, what counts as adequate evidence, which claim should be accepted, and what conclusion can be defended? Writing numerous prompts or sustaining a fluent dialogue with the system does not, by itself, demonstrate that the student made these decisions.

Supported performance and independent competence are also treated as distinct forms of evidence. Producing a successful outcome with AI may demonstrate that the learner can work effectively within a human–AI system. It does not reveal whether the learner can reconstruct the same intellectual structure when AI no longer performs the assessed target operation, or whether a strategy used during the interaction can be applied to a different problem. At the same time, critically directing the system and selecting, reshaping, or rejecting its suggestions in light of one’s own aims are meaningful capabilities in their own right. REAM-AL does not designate one form of performance as valid and dismiss the other. Instead, it asks what kind of inference each can legitimately support.

A fourth assumption is that the developmental value of cognitive offloading cannot be judged simply by how often or how long AI is used. What matters is the role of the offloaded operation in relation to the learning objective. When students use AI to accelerate routine editing, consider alternatives to their own solution, or test an argument against counterexamples, the resources released through offloading may be redirected toward more demanding thought. The picture changes when problem formulation, evidence selection, justification, or inference lies at the heart of what is to be learned, yet these operations are repeatedly left to the system. At that point, support may cease to foster development and begin to substitute for it.

Finally, advanced cognitive capacity is not equated with mature metacognitive control or epistemic readiness. A student with strong domain knowledge may readily detect an obvious factual error while struggling to evaluate a weak line of reasoning presented in fluent and persuasive language. The ability to construct sophisticated prompts likewise says little about whether the learner can determine which evidence is stronger and explain why. REAM-AL consequently treats advanced potential as distinct from, though related to, the capacity to question AI-generated content, recognize the limits of one’s own knowledge, and assume responsibility for epistemic decisions.

### 4.3. Analytical Layers and Focal Constructs

Table 3 clarifies the architecture of REAM-AL by separating contextual conditions, focal constructs, interpretive interaction patterns, and developmental outcomes. This distinction prevents constructs operating at different levels from being treated as though they were equivalent components or sequential stages.

#### 4.3.1. Developmental Epistemic Readiness

Within REAM-AL, developmental epistemic readiness refers to a learner’s current capacity to make sense of an AI-generated claim, question the reasoning and evidence on which it rests, compare alternative explanations, recognize uncertainty, and justify a decision to accept or reject it.

This capacity should not be equated with a general intelligence score, frequency of AI use, or technical skill in prompt construction. How epistemic readiness is expressed during a task may depend on the student’s age, domain knowledge, metacognitive monitoring, habits of critical inquiry, and previous experience with AI. A learner might evaluate system output critically in a familiar domain yet accept fluent answers with little scrutiny when working in an area they know less well. Developmental epistemic readiness is best understood, then, not as a fixed personal trait but as a capacity expressed through the relationship among learner, task, and domain.

Its role in the model is not to produce a binary judgment about whether a student should or should not have access to AI. The more useful question is what kind and intensity of instructional support the learner needs to engage critically with system outputs. Source verification, comparison of evidence, explicit discussion of uncertainty, and teacher feedback can all be differentiated in light of the student’s current level of epistemic readiness.

#### 4.3.2. Cognitive Task Distribution

Cognitive task distribution describes how the operations involved in completing a task are divided between the student and generative AI. These operations may include formulating the problem, searching for information, generating examples, planning, comparing alternatives, selecting evidence, constructing a justification, revising the text, and reviewing the conclusion.

The key issue is not how much the learner uses AI, but which operations are delegated and for what purpose. Asking the system to generate counterexamples to test an independently developed argument creates a different distribution of cognitive work from obtaining both the argument and its supporting rationale from AI. In the first case, the system may serve as a resource that extends the student’s thinking. In the second, it may be carrying out operations central to the intended learning.

Even the same delegated operation can take on a different educational meaning depending on the purpose of the task. If the objective is complex scientific reasoning, using AI to refine the language of a text may free the learner to concentrate on the underlying analysis. If academic writing itself is being assessed, however, linguistic revision may constitute a substantial part of the target competence. Any interpretation of cognitive task distribution must attend both to the nature of the delegated operation and to the task’s explicit learning objective.

When learners retain control over the direction of the task and the criteria by which ideas are judged, delegating selected operations to AI may constitute strategic cognitive offloading. Whether that offloading genuinely expands the student’s thinking cannot be determined from the delegation alone. It must be considered alongside evidence concerning epistemic decision distribution, intellectual ownership, and transfer.

#### 4.3.3. Epistemic Decision Distribution

Within REAM-AL, epistemic decision distribution describes how decisions that shape the direction of knowledge production are distributed among the student, generative AI, and, where relevant, the teacher during an interaction. Cognitive task distribution concerns who performs a particular operation. Epistemic decision distribution asks where authority resides when the problem is framed, sources are judged credible, evidence is deemed sufficient, the central claim is formulated, and a conclusion is selected for defense.

This component draws an important distinction between instrumental prompting skill and epistemic authority. A learner may write numerous prompts or sustain a lengthy exchange with the system without making the central decisions about the problem or the evidence. Assessment should attend less to the number of prompts than to who framed the problem, less to the duration of the interaction than to who evaluated the evidence, and less to the extent of textual revision than to who selected and could defend the central claim.

The distribution of epistemic decisions may also shift as a task unfolds. A student might define the initial problem independently, later accept sources proposed by the system without sufficient scrutiny, and then regain an active role when drawing the conclusion. Epistemic agency is not a stable quality that can be assigned once to the interaction as a whole. It is better understood as a process that may strengthen or recede across different phases of the task.

#### 4.3.4. Intellectual Ownership

In REAM-AL, intellectual ownership concerns whether the student understands the product’s central ideas, problem frame, evidential structure, and conclusions; can distinguish their own contribution from that of AI; and can defend the position they have adopted with reasons. The term is not used here in the legal sense of copyright or formal authorship.

Although intellectual ownership is related to the disclosure of AI use, the two are not equivalent. A learner may give a complete account of when and how AI was used yet remain unable to justify the product’s main claim or explain why particular evidence was selected. Conversely, extensive linguistic assistance from the system does not necessarily eliminate intellectual ownership when the student framed the problem, evaluated the evidence, and can defend the conclusion.

Word counts offer little help in resolving this question. The proportion of human- and AI-generated language in the final text cannot, on its own, establish who owns its underlying intellectual structure. Interaction records, intermediate drafts, the learner’s explanations of key choices, and an oral defense of the work may provide more meaningful evidence of how fully the student understands and endorses what has been produced.

#### 4.3.5. Supported Performance, Independent Competence, and Transfer

REAM-AL separates three evidentiary questions rather than arranging performance into three ordered levels: What can the learner accomplish while working with AI? Can the learner perform the assessed target operation without AI performing it on the learner’s behalf? Can relevant knowledge or strategies be used in a different task?

Supported performance refers to the product a learner creates, or the level of problem-solving achieved, while generative AI contributes within a specified task and context. It may reveal how effectively the student can direct the system, evaluate its outputs, and work within a human–AI arrangement. The AI functions available during the task must therefore be reported.

Independent performance is reserved for conditions in which generative AI does not perform the cognitive or epistemic operation being assessed. This does not require removing every digital tool or all instructional support. Any remaining tools, prompts, or teacher assistance must be specified and must not supply the target operation—such as problem framing, evidence appraisal, justification, or inference—on the learner’s behalf. Performance under such conditions provides evidence about the learner’s independent competence with respect to that operation.

Transfer is a separate dimension concerning change in task demands, not the absence of support. It becomes visible when knowledge, strategies, or forms of justification are applied to a meaningfully different problem rather than merely reproduced in the original format. Transfer may be assessed with AI available or unavailable; the support condition and the target operation must be stated in either case.

Support availability, target-operation independence, task novelty, and assessment timing must therefore be specified separately. An independent task may be familiar, while a transfer task may be completed either with AI support or under conditions in which AI does not perform the target operation. REAM-AL does not collapse these dimensions into a single score. Its concern is to identify which competence each observation can support an inference about and how defensible that inference is.

The theoretical contribution of REAM-AL lies in the distinctions created by connecting these studies. Table 4 shows what each established concept explains, where it leaves the present problem unresolved, and what the model adds. The table does not claim ownership of the source concepts; it identifies the new analytical work performed by their integration.

### 4.4. Contextual Conditions Shaping How the Model Operates

The developmental meaning of a student’s interaction with AI is shaped by the purpose of the task, the characteristics of the domain, the functions available in the system, teacher guidance, assessment design, time pressure, and institutional policies governing AI use. These conditions are not separate components of REAM-AL. They form the contextual layer that influences how relationships among the model’s components take shape.

The learning objective of the task is especially consequential. When students are expected to explore multiple perspectives, having AI generate examples and counterexamples may broaden the range of possibilities they consider. If the aim is to assess their ability to construct an original justification, however, a rationale supplied by the system makes the observed product more difficult to interpret. The same AI function can thus carry very different developmental implications under different learning objectives.

Teacher guidance may alter the interaction just as substantially. An instructional design that requires learners to compare AI-generated claims with other sources, explain why they rejected particular suggestions, and justify their final decisions can help preserve epistemic authority on the student’s side. A design concerned only with the quality of the completed product may leave invisible who performed the central cognitive and epistemic work.

The relationships among the model’s components are neither linear nor unidirectional. Developmental epistemic readiness may influence which operations a learner delegates to AI and how the learner responds to its outputs. Cognitive task distribution is related to epistemic decision distribution, but the two are not interchangeable. A system may perform an operation while the student continues to define its purpose, evaluate the output, and make the final decision. The reverse is also possible: a student may personally revise the prose while adopting the problem frame and central claim from the system. Who performs an operation and who retains authority over it must be examined separately.

Epistemic decision distribution is expected to bear on intellectual ownership, although making a decision does not necessarily mean that the student understands its rationale. Evidence of ownership must consequently extend beyond a record of who chose what. The learner’s ability to explain, reconstruct the reasoning behind, and defend the product also matters. Independent reconstruction and transfer may add a further layer of evidence by showing whether the claimed ownership rests on an understanding that can be sustained, rather than on the student’s account alone.

How repeated, structured human–AI interactions might alter a learner’s epistemic readiness over time has yet to be demonstrated directly. REAM-AL treats this as an empirical question. Opportunities for critical evaluation may strengthen such readiness, whereas repeatedly assigning central decisions to the system may restrict the learner’s opportunities to practise epistemic judgment independently.

Assessment design determines, to a considerable extent, which elements of the interaction become visible. Process records, intermediate drafts, student explanations, oral defenses, and tasks completed under reduced-support conditions can provide converging evidence about cognitive task distribution, epistemic authority, and intellectual ownership. A score assigned only to the final product is unlikely to reveal how that performance was produced.

**Figure 3:**
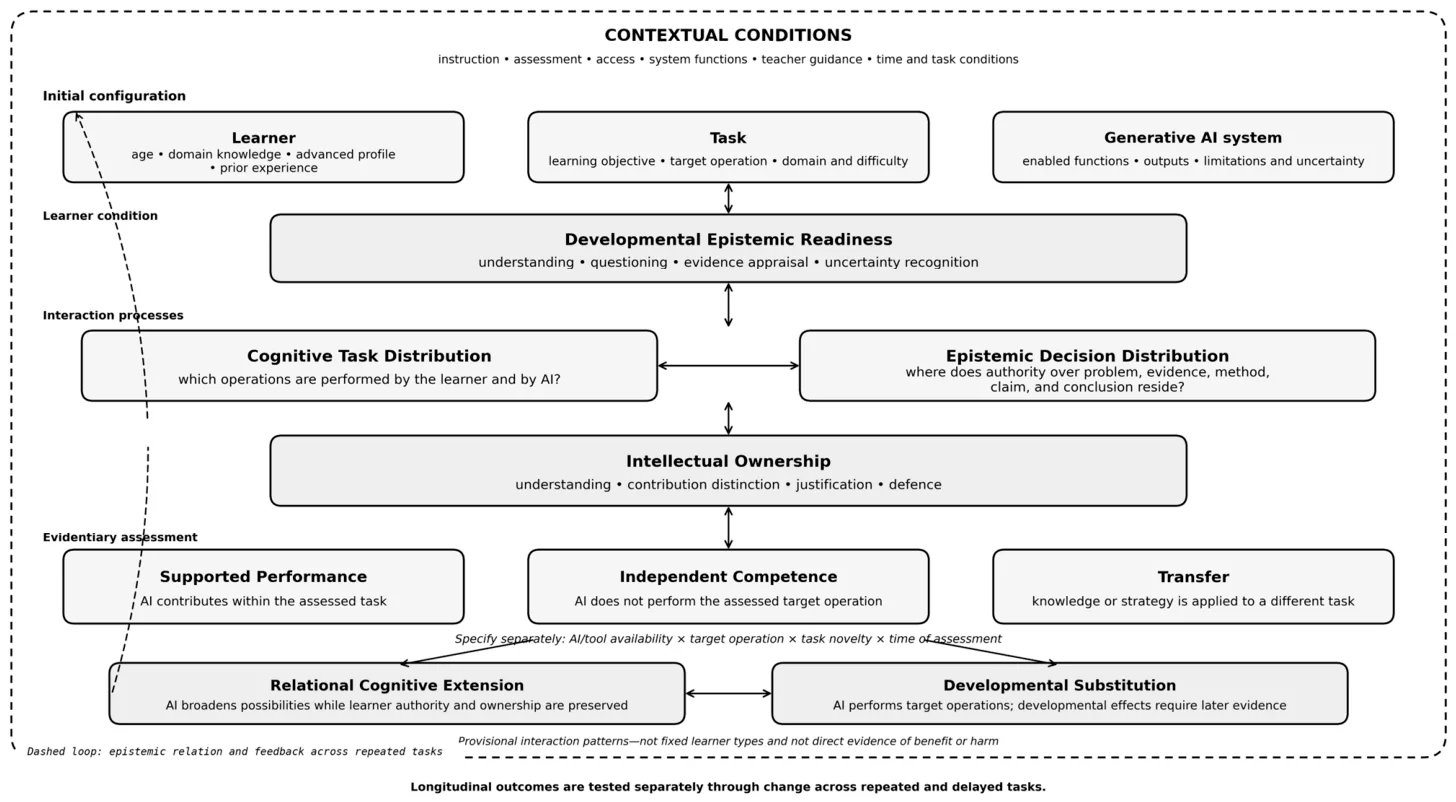
Dynamic structure of the Relational Epistemic Agency Model for Advanced Learners (REAM-AL). Note. Double-headed arrows indicate mutually informing relationships, not a fixed causal order. Within the evidentiary layer, independent competence requires that AI not perform the assessed target operation; transfer concerns performance on a meaningfully different task and may be examined with or without AI. Relational cognitive extension and developmental substitution are provisional task-level interpretations rather than fixed learner types or direct developmental outcomes. The dashed pathway indicates that experience in one interaction may alter the learner’s starting conditions in later tasks.

Figure 3 presents REAM-AL as a set of mutually informing analytical layers rather than a sequence of developmental stages. The initial learner–task–AI configuration is situated within contextual conditions. Developmental epistemic readiness, cognitive task distribution, epistemic decision distribution, intellectual ownership, and performance evidence are examined in relation to one another. Within the evidentiary layer, AI availability, the assessed target operation, task novelty, and assessment timing are specified separately; independence and transfer are not treated as successive levels. The resulting configuration supports a provisional interpretation of relational cognitive extension or developmental substitution. Longer-term benefit, no effect, or cost is assessed separately through change across repeated and delayed tasks.

### 4.5. Interaction Patterns and Their Developmental Implications

REAM-AL uses the configuration of its five focal constructs to interpret two possible interaction patterns: relational cognitive extension and developmental substitution. These patterns describe how cognitive and epistemic work is organized within a particular student–task–AI interaction. They are not stable learner categories, and neither pattern by itself establishes a beneficial or harmful developmental effect.

The patterns are neither mutually exclusive poles nor a fixed sequence. An interaction may move between them as the task unfolds. Their developmental implications must be evaluated through change over time: baseline performance; performance with AI; immediate and delayed performance under conditions in which AI does not perform the assessed target operation; and transfer to tasks that vary in novelty, with AI availability reported separately. A difference between AI-supported and later independent performance shows that the performance conditions differ; it does not, by itself, show that learning was impaired.

#### 4.5.1. Relational Cognitive Extension

Within REAM-AL, relational cognitive extension describes an interaction in which generative AI expands the learner’s intellectual possibilities without replacing the learner’s thinking. Epistemic authority and intellectual ownership remain with the student.

In such an interaction, the learner continues to frame the problem, evaluate evidence, select the central claim, and defend the conclusion. AI contributes functionally by offering alternative explanations, counterexamples, comparisons, or preliminary organizational options. The student’s ability to question these outputs, reshape them according to independent criteria, reject inadequate suggestions, and explain the choices made provides important evidence of this pattern.

An interpretation of relational cognitive extension becomes more persuasive when improvement in product quality is accompanied by the learner’s ability to reconstruct the underlying intellectual structure with reduced support or apply it to a new problem. This does not require the student to perform every operation involved in the task alone. The decisive issue is whether epistemic authority over the direction of thought—and intellectual ownership of the product’s central structure—remains with the learner despite the operations delegated to technology.

Strategic cognitive offloading is one mechanism through which relational cognitive extension may occur, but the two concepts are not synonymous. Strategic offloading refers to the deliberate delegation of selected operations to an external resource. Relational cognitive extension describes the broader developmental pattern that may emerge for the learner from that arrangement.

#### 4.5.2. Developmental Substitution

Developmental substitution describes a task-level interaction pattern in which AI performs operations that the learner is expected to practise or demonstrate as part of the learning objective. If the task is intended to develop problem formulation, evidence appraisal, justification, or inference, assigning most of that work to the system may make the supported product a poor indicator of the learner’s independent contribution. The construct concerns the organization of activity during the task; it does not by itself establish that the learner has lost competence or failed to learn.

A polished product can therefore coexist with developmental substitution, but the latter consequence remains an empirical question. AI may supply a solution that the learner subsequently examines, questions, and internalizes, functioning much like an interactive worked example. Alternatively, the learner may accept the solution without reconstructing its reasoning. Distinguishing these possibilities requires a baseline and subsequent independent measures, not merely a comparison between the supported product and one post-support score.

That distance can be particularly difficult to recognize in advanced learners. Strong domain knowledge, verbal fluency, and rapid pattern recognition may allow a student to assemble AI-generated material into a coherent and persuasive whole. The product may consequently appear advanced even when the system has performed a substantial share of the task’s central operations. A product score alone cannot establish that the student has attained independent advanced competence. The distribution of work during the process and the performance sustained under reduced-support conditions must also be examined.

#### 4.5.3. Identifying the Patterns

No single indicator separates relational cognitive extension from developmental substitution. Process evidence should show how cognitive operations and epistemic decisions were distributed, whether the learner could distinguish and defend their contribution, and how the learner engaged with system output. Performance evidence should vary three dimensions deliberately: the availability of support (full, faded, or removed), the novelty of the task (same or familiar task, structurally equivalent task, and near or far transfer), and the time of measurement (immediate or delayed). Together, these data permit a more defensible interpretation of both the interaction pattern and its developmental consequences.

Even converging indicators should not be used mechanically. A successful oral defense supports an inference about understanding and intellectual ownership, but it does not show that the learner independently completed every stage of the work. Weak transfer may reflect developmental substitution, but it may also arise from insufficient prior knowledge, excessive transfer distance, or features of the assessment. Conversely, improvement from baseline after an AI-supported episode may indicate learning even when the later independent score remains below the supported score. Claims about benefit, no effect, or harm therefore require an explicit comparison over time and, where possible, an appropriate comparison condition.

### 4.6. Theoretical Propositions Derived from REAM-AL

The conceptual structure of REAM-AL gives rise to eight expectations that can be examined empirically.

**Proposition 1.** 
*The high quality of an AI-supported product will not, on its own, constitute evidence of advanced independent competence. Such an inference also requires evidence that the learner made the central epistemic decisions and can demonstrate the relevant competence independently or in a new task.*

**Proposition 2.** 
*Learners with greater developmental epistemic readiness will be more likely to notice not only factual errors in AI output, but also weak reasoning and unresolved uncertainty. Their decisions to accept or reject system suggestions will rely more on the quality of the evidence than on the surface persuasiveness of the response.*

**Proposition 3.** 
*Interactions in which students retain responsibility for formulating the problem, evaluating evidence, and constructing a justification will be more likely to display relational cognitive extension. When these operations are largely performed by the system, the interaction will be more likely to display developmental substitution. Whether either pattern predicts later learning must be tested against baseline, delayed independent performance, and transfer rather than inferred from process organization alone.*

**Proposition 4.** 
*Prompt frequency and time spent interacting with the system may indicate intensity of use, but they will reveal relatively little about epistemic agency. More informative indicators will include how the learner framed the problem, the criteria used to weigh evidence, how the central claim was selected, and whether the conclusion could be defended.*

**Proposition 5.** 
*Intellectual ownership will be more closely associated with the learner’s ability to explain the product, justify key choices, reconstruct its intellectual structure under reduced-support conditions, and transfer that structure to another task than with either the proportion of text written by the learner or the disclosure of AI use.*

**Proposition 6.** 
*Structured tasks that require students to verify sources, compare competing rationales, monitor their own thinking, and defend their work orally will make epistemic authority and intellectual ownership more visible than tasks in which only the final product is scored.*

**Proposition 7.** 
*The pattern displayed by a learner will not remain fixed across situations. As the purpose of the task, available AI functions, instructional guidance, and level of support change, the same student may exhibit interaction patterns that move closer either to relational cognitive extension or to developmental substitution.*

**Proposition 8.** 
*Across repeated tasks, interaction patterns will predict developmental change only when prior competence and the timing, novelty, and support conditions of later assessments are taken into account. Recurrent relational cognitive extension is expected to be associated with maintained or improved independent reconstruction and transfer. Recurrent developmental substitution may restrict opportunities for practice, but it will count as evidence of developmental cost only if later independent or transfer performance is lower than an appropriate baseline or comparison trajectory.*

These propositions cannot be tested by comparing supported and post-support product scores alone. Designs should cross support availability (full, faded, or removed) with task novelty (same, structurally equivalent, near-transfer, or far-transfer tasks) and time (immediate or delayed assessment). Process evidence may include prompt–response sequences, screen records, draft histories, student explanations, think-aloud data, and oral defenses. Developmental claims additionally require baseline measures and, where feasible, comparison conditions. Proposition 8 requires longitudinal evidence because it concerns change across repeated interactions rather than an immediate effect observable within a single session.

The model also generates results that would count against its central claims. The proposed role of developmental epistemic readiness would be weakened if measures of evidence appraisal and uncertainty detection failed to predict students’ acceptance or rejection of AI outputs after domain knowledge was controlled. The distinction between interaction intensity and epistemic agency would be weakened if prompt counts and time-on-system predicted independent reasoning as well as, or better than, coded decision authority. The proposed role of intellectual ownership would be challenged if explanation and defense were unrelated to later independent reconstruction or transfer. Most importantly, the distinction between relational cognitive extension and developmental substitution would have limited developmental value if process classifications did not predict change beyond baseline competence across delayed and transfer tasks. These possibilities make revision or rejection of particular relationships part of the intended empirical program.

### 4.7. Scope and Boundaries of the Model

The scope of REAM-AL is deliberately bounded. It was developed to interpret the performance of advanced children and adolescents in generative AI-supported cognitive tasks from developmental and epistemic perspectives. It is not a general theory of human–AI interaction validated across all AI systems, student populations, or age groups. Nor does it seek to explain the learner or the system in isolation. Its object of analysis is the interaction pattern that develops between them within a particular task and context.

Although childhood and adolescence are central to the model, much of the available empirical research comes from university students and adults. The revised evidence-proximity scheme prevents these studies from being treated as direct evidence about advanced children. Direct advanced child/adolescent evidence and gifted university evidence are reported separately from general developmental studies, adult mechanism evidence, and theoretical extrapolation. The adult literature identifies mechanisms and research designs worth testing; it does not verify REAM-AL’s population-specific expectations. The model’s distinctive claims—particularly that advanced product-building capacities may obscure who performed the central reasoning, and that the developmental meaning of delegation may change with domain progression—must be examined directly across ages and fields.

Using REAM-AL as a diagnostic or scoring instrument would exceed its intended purpose. Its components have not yet been validated as psychometric dimensions, and relational cognitive extension and developmental substitution were not designed as enduring labels to be attached to individual students. The next step is to develop observable indicators for these constructs. Researchers will then need to determine whether the indicators can be coded reliably by different raters and whether they retain comparable meanings across domains and task types.

Empirical tests should also resist treating the technology as a fixed, context-free tool. Reports need to specify the name and version of the system, the functions enabled, prompting and feedback conditions, duration of use, and the nature of teacher guidance. Features such as accuracy, memory, personalization, explainability, and user control may differ across versions. Access to technology, digital competence, language of use, socioeconomic resources, and school policies may likewise change what the same task means for different learners. REAM-AL treats these influences not as core components, but as contextual conditions shaping the interpretation of the interaction. The proposed relationships are theoretical expectations awaiting investigation across different ages, domains, tasks, and system conditions, not causal conclusions that have already been established.

## 5. Discussion

The central issue is how far an advanced product created with AI support can justify inferences about the learner who produced it. Research in gifted education identifies promising applications in personalization, differentiation, inquiry, writing, and mentoring, but the direct evidence remains small, heterogeneous, and largely short-term (Almumen & Jouhar, 2025; Alsamani & Alsamiri, 2026; Guilbault et al., 2025). The question is therefore not whether such uses are promising, but what kind of competence the resulting performance represents.

REAM-AL addresses this problem by separating technological affordance from development within the learner. AI can widen access to alternatives, place competing explanations side by side, and accelerate routine operations. Those possibilities do not become learning gains automatically. The student must still weigh outputs, reject inadequate suggestions, retain authority over the problem and evidence, and explain the intellectual structure that emerges. Stronger evidence of development appears when knowledge and strategies encountered during supported work can be reconstructed after the system is withdrawn and used in a different problem.

The performance–learning distinction developed in Section 2 becomes central here. Studies with university samples show that product quality, retained knowledge, and transfer can follow different trajectories (Barcaui, 2025; Fan et al., 2025). Their value for REAM-AL is methodological rather than population-specific: they show why an advanced-looking product cannot serve as the sole outcome measure.

Extended-mind and distributed-cognition perspectives provide a theoretical basis for resisting the attribution of AI-supported work solely to the isolated learner. When cognitive activity is organized across people, tools, and representations, the resulting system may achieve a level of performance unavailable to the individual working alone (Clark & Chalmers, 1998; Hollan et al., 2000; Hutchins, 1995). Success at the system level, however, does not establish equivalent development in the human participant. REAM-AL does not discount jointly produced performance. It challenges the assumption that such performance provides the same evidence as the learner’s independent competence.

The literature on cognitive offloading brings the possible divergence between joint performance and individual development into sharper focus. An external resource may reduce cognitive demand and allow limited internal resources to be redirected toward more complex operations; offloading is not inherently a developmental loss (Risko & Gilbert, 2016). Nor does improved performance during tool use demonstrate that internal learning, or competence available without the tool, has advanced to the same extent (Grinschgl & Neubauer, 2022). The consequential question is not how much AI was used, but which operation was delegated and in relation to what learning objective.

Context can change the developmental meaning of the same system function. When the goal is scientific reasoning, delegating linguistic refinement may allow the learner to devote more attention to conceptual relationships. If the objective is to develop academic writing, that same choice may assign a substantial part of the target competence to the system. Asking for counterexamples or alternative explanations can open space for testing an independently formed argument. Delegating problem formulation, evidence selection, and justification in their entirety may instead displace the thinking the task was designed to cultivate. REAM-AL conceptualizes the former pattern as relational cognitive extension and the latter as developmental substitution.

Advanced learners’ strengths may make substitution unusually difficult to detect. A student with extensive domain knowledge, verbal fluency, and strong organizational skills may assemble AI-generated fragments into a coherent product that appears well beyond age expectations. Yet the system may still have performed the central work of formulating the problem, evaluating the evidence, or constructing the justification. An advanced-looking product cannot simply be equated with advanced independent competence. Whether this masking effect occurs among advanced children, and under what conditions, remains an empirical question and one of REAM-AL’s central testable propositions.

A further contribution of the model lies in separating cognitive capacity from epistemic readiness. Metacognitive monitoring and control continue to develop throughout childhood and may influence how students make use of instructional support (Bayard et al., 2021; O’Leary & Sloutsky, 2017). Urban et al. (2025) found that ChatGPT could increase the inclusion of correct information without significantly reducing reliance on erroneous information generated by the system. Xu et al. (2025), meanwhile, reported that explicit metacognitive support improved task strategies and self-evaluation but did not produce significant between-group differences in achievement scores. Read together, these results make an important point: access to the system does not generate critical use on its own. Learners may also need to be taught how to question what it produces.

The limited evidence from gifted students points in the same direction. Kang and Lee (2023) found that gifted identification did not ensure consistently high-level questioning during a ChatGPT-supported physics activity. Because the study was small and domain-specific, it is used here to motivate—not confirm—the distinction between cognitive capacity and epistemic readiness.

Metacognitive control helps explain how students monitor and regulate their own thinking, but it cannot tell us who directs the production of knowledge. In work on epistemic agency, responsibility for knowledge goals, questions, and evidence occupies a central place (Damşa et al., 2010; Scardamalia, 2002). A high number of prompts, an extended conversation with the system, or substantial editing of generated text cannot establish that epistemic responsibility remained with the learner. An intensive user who accepts an AI-framed problem and its evidence without scrutiny may exercise less epistemic authority than a student who uses less support but evaluates alternatives according to independently held criteria. REAM-AL accordingly focuses on how decisions about the problem, method, evidence, and conclusion are distributed, rather than on volume of use.

Where those decisions reside has direct implications for intellectual ownership and assessment validity. Detecting AI involvement in a text does not reveal whether the student understands the text or constructed its underlying intellectual structure. Kofinas et al. (2025) found that evaluators could not reliably distinguish work involving AI from work without such involvement; even suspicion of AI use could affect their assessment behavior. Van Vlasselaer et al. (2026) likewise showed that detector performance varies with the system used and the way the text was produced. The validity question cannot be reduced to “Who wrote the text?” It must also address the learner’s intellectual relationship with what has been submitted.

Intellectual ownership in REAM-AL extends well beyond the proportion of words written by a human. The learner should understand the central ideas and organization of the evidence, distinguish personal contributions from those of the system, and defend the adopted conclusion with reasons. Ebrahimzadeh et al. (2026) draw attention to the threat to co-authorship integrity and assessment validity when students incorporate AI-generated material they do not understand. REAM-AL adds a developmental test to that discussion: Can the learner merely defend the present product, or can the same intellectual structure be reconstructed with less support and carried into another problem?

This distinction avoids treating AI-supported performance as either wholly valid or wholly invalid. Such work may provide strong evidence of purposeful participation in a human–AI system while offering weaker evidence of independent writing, reasoning, or justification (Swiecki et al., 2022). The relevant inference must therefore be stated explicitly rather than compressed into a single score.

Here REAM-AL adds a further layer to theories of talent development. Such theories understand the transformation of initial high capacity into domain-specific competence as a process shaped over time by high-quality instruction, domain knowledge, psychosocial skills, and developmental opportunities (Gagné, 2015; Olszewski-Kubilius et al., 2015; Subotnik et al., 2011). Relational and systemic perspectives similarly locate performance in the interaction among individual characteristics, tasks, environments, and learning resources (Barab & Plucker, 2002; Ziegler et al., 2017). Generative AI is no ordinary feature of that environment: it can contribute content, arguments, and justification directly to the observed performance. The problem is no longer only how capacity becomes visible in context. It is also how the cognitive and epistemic contributions embedded in the product can be disentangled.

Acknowledging this contribution does not require treating the human and AI as epistemic equals. A system may perform substantial functional work within a task, but it is not regarded here as a subject that understands its claims as a human would or bears educational and epistemic responsibility for them (Bender et al., 2021; Rossi et al., 2026; Shanahan, 2024). The question introduced at the outset—whether thinking with generative AI produces a different form of intelligence—is not answered by positing an ontologically new kind of human intelligence. What has changed are the conditions under which advanced performance is produced, observed, assessed, and attributed to the learner. Rather than naming a new intelligence, REAM-AL invites a reconsideration of the relationship between relational performance and individual development.

Turning the model into an empirical research program will require observable indicators for each of its five components. Screen and interaction records, prompt–response sequences, draft histories, student explanations, and think-aloud protocols may help trace the distribution of cognitive tasks and epistemic decisions. Intellectual ownership cannot be inferred from disclosure alone. It needs to be examined through the learner’s ability to explain the product, defend evidential choices, distinguish personal contributions from those of the system, and reconstruct a comparable line of reasoning in a new task. Any coding framework developed for these purposes will also require evidence of interrater reliability and stability across different tasks.

Research testing the developmental propositions should cross rather than collapse the relevant design dimensions: AI availability, whether AI performs the target operation, task novelty, and time of assessment. Product quality, accuracy, quality of justification, delayed retention, and transfer success should remain separate outcomes. Single-session experiments can provide a useful starting point, but the effects of metacognitive guidance, source verification, teacher feedback, and gradually faded support will require longitudinal investigation. Such research should not begin from the assumption that AI necessarily produces either development or loss. The more productive question is under which combinations of learner, task, and system conditions each developmental pattern emerges.

## 6. Conclusions and Recommendations

This article began with a question: What can an advanced product created with generative AI tell us about a learner’s independent competence? The literature presents two rather different pictures. On one side are emerging opportunities for personalization, differentiation, writing, creativity, and mentoring. On the other is evidence that success on supported products does not always coincide with retention, independent performance, or transfer. What has been missing is a framework capable of bringing these lines of evidence together while accounting for the developmental characteristics of advanced learners, their authority over epistemic decisions, and the conditions under which their performance is assessed.

REAM-AL responds to that gap by bringing developmental epistemic readiness, cognitive task distribution, epistemic decision distribution, intellectual ownership, and the supported performance–independent competence–transfer relationship into a single model. Its novelty does not lie in proposing a new kind of intelligence or a set of fixed categories into which students can be placed. Instead, it offers a language for explaining how two outwardly similar products may emerge from very different cognitive and epistemic processes. This makes it possible to ask which aspects of relationally produced performance can support an inference about the learner’s competence, and under what conditions.

Relational cognitive extension and developmental substitution should not be mistaken for fixed poles separating “beneficial” from “harmful” AI use. In the first pattern, the system expands the learner’s intellectual possibilities while authority over the problem, evidence, and conclusion—and ownership of the product’s central structure—remains with the student. In the second, AI performs operations central to the learning objective, allowing the supported product to present an inflated picture of the learner’s independent development. Because these patterns characterize interactions rather than enduring properties of students, the same learner may occupy a different position when the task or support conditions change.

For education, the implication is to move beyond the choice between unrestricted acceptance and complete prohibition. The intellectual operations students are expected to develop and demonstrate must be identified first. Decisions about which system functions may be used can then be made in relation to those aims. Advanced learning potential should not be equated with readiness for unstructured AI use. Verifying sources, comparing alternative explanations, recognizing uncertainty, and accepting or rejecting system suggestions with reasons all need to become explicit instructional goals.

Assessment practices require a similar shift. Neither a final-product score nor the output of an AI detector should serve as the sole basis for judgment. In high-stakes decisions involving admission to gifted programs, level placement, or scholarships, an AI-supported product should be accompanied by complementary evidence such as process records, a brief oral defense, reconstruction under reduced-support conditions, and a transfer task. The purpose is not to penalize students for using AI. It is to establish what competence the observed performance can defensibly be taken to represent. Disclosure of AI use is necessary, but it cannot substitute for intellectual ownership demonstrated through understanding and defense of the work.

School policies should reflect the same principle. Rather than offering only lists of prohibitions and detection procedures, they should specify in advance which system functions are permitted for particular tasks, how the learner’s contribution will be made visible, and what complementary evidence will be collected. Teacher preparation cannot stop at technical AI literacy. It must also address cognitive task distribution, epistemic authority, intellectual ownership, and assessment validity. Questions of equity matter here as well. Students do not have equal access to paid features, devices, different AI systems, or adult guidance, and those disparities need to be considered when their performance is interpreted.

REAM-AL is not yet a validated developmental theory or a ready-to-use assessment instrument. Its present value is more modest, but still consequential: it interrupts the rapid attribution of AI-supported products directly to student competence and redirects attention to the cognitive–epistemic arrangement through which those products were created. The next step is a program of experimental, mixed-methods, and longitudinal research with advanced children and adolescents. Such work will need to establish how the model’s components can be observed and identify the conditions under which relational cognitive extension and developmental substitution emerge.

## Figures and Tables

**Table 1:** Priority levels used in the preliminary assessment of sources.

Priority Level	Definition	Role in the Assessment
P1—Core Candidate	Records directly related to advanced learners or to the core mechanisms of REAM-AL	Given first priority for full-text review and assessment of claim–evidence alignment
P2—Supporting Candidate	Records showing a strong conceptual relationship with multiple components of the model	Used to explain relationships among components and to support the interpretation of direct evidence
P3—Contextual or Supplementary Source	Records that could contribute indirectly to a specific component, mechanism, or contextual condition of the model	Considered as complementary sources in areas where direct evidence was limited
P4—Low Relevance to Scope	Records with limited relevance to the research problem or the model’s conceptual structure	Not prioritized for detailed review

Note. The priority levels do not indicate the methodological quality of the studies or the strength of their evidence. Classification at this stage was based solely on conceptual proximity as judged from titles and abstracts. Final assessment drew on the full text, research design, sample, the extent to which the reported findings supported the relevant claim, and bibliographic verification.

**Table 2:** Evidence-proximity categories used in developing REAM-AL.

Evidence-Proximity Category	Scope	Use in the Model
Direct Advanced Child/Adolescent Evidence	Research conducted with gifted or otherwise advanced children and adolescents	Used as the closest empirical basis for target-population claims; design and domain limits remain explicit
Gifted or Advanced University Evidence	Research conducted with university students identified as gifted, advanced, or high-achieving	Used to examine advanced-learner experiences while acknowledging developmental and institutional differences from school-age learners
General Child/Adolescent Evidence	Studies involving children or adolescents without an advanced-learner criterion	Used to inform developmental mechanisms such as metacognitive monitoring; not treated as evidence of gifted-group specificity
University/Adult Mechanism Evidence	Research with general university or adult samples examining offloading, learning, retention, transfer, ownership, or assessment	Used to identify mechanisms and design variables that require direct testing in advanced children and adolescents
Theoretical Extrapolation	Foundational theories, assessment standards, formal frameworks, and relationships generated through the present synthesis	Used to define constructs and derive propositions; clearly distinguished from population-specific empirical findings

Note. Evidence proximity is distinct from methodological quality. A well-designed adult study may offer strong evidence for a general mechanism while remaining indirect evidence for advanced children. Conversely, a small target-population study is more proximal but may still support only a cautious inference. Both dimensions were considered when claims were formulated.

**Table 3:** Architecture of REAM-AL.

Analytical Level	Construct or Element	Central Question	Status in the Model
Initial configuration	Learner, task, AI system, and instructional/assessment context	Under what conditions does the interaction occur?	Contextual conditions; not focal constructs
Learner condition	Developmental epistemic readiness	What can the learner understand, question, compare, and evaluate?	Focal construct 1
Interaction process	Cognitive task distribution	Who performs which cognitive operation, and for what purpose?	Focal construct 2
Interaction process	Epistemic decision distribution	Who holds authority over the problem, evidence, method, claim, and conclusion?	Focal construct 3
Epistemic relation	Intellectual ownership	Does the learner understand, distinguish, justify, and defend the product’s central structure?	Focal construct 4
Evidentiary assessment	Supported performance–independent competence–transfer relationship	What does performance under changing support, task, and time conditions justify inferring?	Focal construct 5
Interpretive pattern	Relational cognitive extension or developmental substitution	Which pattern best fits the converging process and performance evidence?	Provisional task-level interpretation; not a learner type or direct outcome
Developmental consequence	Change in independent competence, transfer, self-regulation, and epistemic responsibility	What changes across repeated interactions and over time?	Longitudinal outcome to be tested

Note. The five focal constructs are distributed across four analytical layers. Relational cognitive extension and developmental substitution are provisional interaction patterns inferred from converging evidence; longer-term benefit, no effect, or harm must be established through change over time.

**Table 4:** From established concepts to the distinctive extensions proposed by REAM-AL.

Established Concept	What It Explains	Unresolved Issue in Advanced Learner–GenAI Interaction	REAM-AL Extension and Linked Proposition
Extended and distributed cognition	How cognitive activity can be organized across people, tools, and representations	Successful joint performance does not show who retained authority over the problem and evidence or what the learner can later sustain	Epistemic decision distribution separates functional contribution from decision authority (Propositions 3–4)
Cognitive offloading	Why external resources can reduce or reorganize task demands	It does not by itself distinguish delegating a routine operation from delegating an operation central to talent development, or establish the later learning effect	Developmental substitution identifies task-level replacement of target operations while reserving claims about benefit or cost for longitudinal evidence (Propositions 3 and 8)
Metacognitive monitoring and control	How learners judge and regulate their own cognition	Accurate self-monitoring does not establish readiness to evaluate fluent AI claims or locate authority over knowledge decisions	Developmental epistemic readiness links monitoring with the evaluation of evidence, uncertainty, and AI-generated reasoning (Proposition 2)
Epistemic agency	Learners’ responsibility for shared knowledge goals and inquiry direction	Visible interaction or prompting does not reveal where authority over problem, method, evidence, and conclusion resides	Decision-point analysis traces epistemic authority across phases of an AI-supported task (Propositions 3–4)
Assessment validity and transfer	What evidence is needed to support interpretations of performance and learning	A final AI-supported product may represent the joint system while being attributed to the learner alone	Intellectual ownership and the support–task novelty–time framework separate joint performance, independent competence, and transfer (Propositions 1, 5–6, and 8)

Note. The propositions are theoretical expectations. Their numbering refers to Section 4.6.

## Data Availability

This article reports a theoretical model-development study based on published literature. No new empirical data were created or analyzed. The documented search strategies and record counts are provided in Supplementary Table S1.

## Supplementary Materials

The following supporting information can be downloaded at: https://www.mdpi.com/article/10.3390/jintelligence14100247/s1. Supplementary Table S1: Database Search Strategies and Record Counts.
